# High-resolution mapping of *SrTm4*, a recessive resistance gene to wheat stem rust

**DOI:** 10.1007/s00122-023-04369-z

**Published:** 2023-04-27

**Authors:** Hongna Li, Jing Luo, Wenjun Zhang, Lei Hua, Kun Li, Jian Wang, Binyang Xu, Chen Yang, Guiping Wang, Matthew N. Rouse, Jorge Dubcovsky, Shisheng Chen

**Affiliations:** 1 National Key Laboratory of Wheat Improvement, Peking University Institute of Advanced Agricultural Sciences, Shandong Laboratory of Advanced Agriculture Sciences in Weifang, Weifang, 261325 Shandong China; 2 Department of Plant Sciences, University of California, Davis, CA95616 USA; 3 US Department of Agriculture-Agricultural Research Service, Cereal Disease Laboratory and Department of Plant Pathology, University of Minnesota, St. Paul, MN 55108 USA; 4 Howard Hughes Medical Institute, Chevy Chase, MD 20815 USA; 5 Triticeae Research Institute, Sichuan Agricultural University, Chengdu, 611130 China

## Abstract

**Key message:**

The diploid wheat recessive stem rust resistance gene *SrTm4* was fine-mapped to a 754-kb region on chromosome arm 2A^m^L and potential candidate genes were identified.

**Abstract:**

Race Ug99 of *Puccinia graminis* f. sp. *tritici* (*Pgt*), the causal agent of wheat stem (or black) rust is one of the most serious threats to global wheat production. The identification, mapping, and deployment of effective stem rust resistance (*Sr*) genes are critical to reduce this threat. In this study, we generated *SrTm4* monogenic lines and found that this gene confers resistance to North American and Chinese *Pgt* races. Using a large mapping population (9522 gametes), we mapped *SrTm4* within a 0.06 cM interval flanked by marker loci *CS4211* and *130K1519*, which corresponds to a 1.0-Mb region in the Chinese Spring reference genome v2.1. A physical map of the *SrTm4* region was constructed with 11 overlapping BACs from the resistant *Triticum monococcum* PI 306540. Comparison of the 754-kb physical map with the genomic sequence of Chinese Spring and a discontinuous BAC sequence of DV92 revealed a 593-kb chromosomal inversion in PI 306540. Within the candidate region, we identified an L-type lectin-domain containing receptor kinase (*LLK1*), which was disrupted by the proximal inversion breakpoint, as a potential candidate gene. Two diagnostic dominant markers were developed to detect the inversion breakpoints. In a survey of *T. monococcum* accessions, we identified 10 domesticated *T. monococcum* subsp. *monococcum* genotypes, mainly from the Balkans, carrying the inversion and showing similar mesothetic resistant infection types against *Pgt* races. The high-density map and tightly linked molecular markers developed in this study are useful tools to accelerate the deployment of *SrTm4*-mediated resistance in wheat breeding programs.

**Supplementary Information:**

The online version contains supplementary material available at 10.1007/s00122-023-04369-z.

## Introduction

Common wheat (*Triticum aestivum* L., 2*n* = 6*x* = 42, AABBDD) is a major crop used for consumption by humans and domestic animals in many areas of the world and further increases in production are required to accommodate a growing human population. However, these increases are limited by losses generated by pathogens, with fungal pathogens among the major constraints for increasing global wheat production (Figueroa et al. 2018). Among these pathogens, *Puccinia graminis* f. sp. *tritici* (*Pgt*) causes stem (or black) rust of wheat and is potentially a devastating disease worldwide. Severe wheat stem rust epidemics occurred in the United States and Europe in the nineteenth and early twentieth centuries causing large yield losses (Roelfs 1978; Zadoks 1967). Due to efforts to eradicate the alternate host barberry (*Berberis vulgaris* L.) and breed for resistant wheat varieties, this disease was successfully controlled over the past several decades (Peterson 2001; Roelfs 1982; Singh et al. 2015).

However, stem rust re-emerged as a major concern with the appearance of *Pgt* race TTKSK (also known as Ug99) in Uganda in 1998 (Pretorius et al. 2000). Ug99 was the first *Pgt* race to defeat the widely deployed stem rust resistance gene *Sr31* (Jin et al. 2007; Pretorius et al. 2000). Subsequently, Ug99 and its derivatives spread throughout the major wheat-growing countries of eastern and southern Africa (Pretorius et al. 2020; Shahin et al. 2020; Singh et al. 2015) and into the Middle East (Nazari et al. 2009, 2021), and gained virulence to additional *Sr* genes, including *Sr24* (Jin et al. 2008), *Sr36* (Jin et al. 2009), and *SrTmp* (Newcomb et al. 2016).

In recent years, other highly virulent *Pgt* races outside the Ug99 race group, such as TKTTF, TRTTF, RRTTF and TTRTF, were detected in wheat stem rust outbreaks. Race TKTTF was responsible for severe stem rust epidemics in Ethiopia in 2013/14 on the Ug99 resistant wheat cultivar “Digalu” (Olivera et al. 2015), and was subsequently found in more than ten countries, including Ethiopia, Azerbaijan, Egypt, Iraq, Iran, Sudan, Lebanon, Turkey, Germany, Sweden, Denmark and the UK (Lewis et al. 2018; Patpour et al. 2017). Race TRTTF and the closely related race RRTTF were detected in Pakistan, Yemen, Ethiopia, and Ecuador (Barnes et al. 2018; Fetch et al. 2016), and were virulent to at least three *Sr* genes (*Sr36*, *SrTmp*, and *Sr1RS*^*Amigo*^) that are effective against race TTKSK (Olivera et al. 2012). Another *Pgt* race of concern is TTRTF, which caused a severe epidemic of stem rust on thousands of hectares of durum and bread wheat in Southern Italy in 2016 (Bhattacharya 2017). This race overcame the resistance provided by *Sr13b*, *Sr21*, *Sr35*, *Sr45*, *Sr50* and several other *Sr* genes (Barnes et al. 2018; Bhattacharya 2017; Patpour et al. 2020), and was recently reported in more countries, including Hungary, Egypt, Ethiopia, Eritrea and Iran (Olivera et al. 2019; Patpour et al. 2020; Tesfaye et al. 2020). Additionally, several *Pgt* races caused large scale wheat stem rust outbreaks in Northern Kazakhstan and Western Siberia generating yield losses in more than one million hectares of spring wheat (Rsaliyev et al. 2020; Skolotneva et al. 2020). The recent evolution and spread of new virulent *Pgt* races have prompted widespread efforts to identify new *Sr* genes and to develop new wheat cultivars with durable resistance.

So far, over 60 *Sr* genes (*Sr1-Sr63*) have been assigned official designations (Mago et al. 2022; Yu et al. 2022), among which a significant proportion were introgressed into wheat from wild relatives (Singh et al. 2015). *Triticum monococcum* (2*n* = 2*x* = 14, A^m^A^m^), is commonly known as einkorn wheat. This diploid species is closely related to *Triticum urartu* (A^u^A^u^), the A-genome donor of polyploid wheat (Dvorak et al. 1988). *Triticum monococcum* has contributed five *Sr* genes, including *Sr21* (Chen et al. 2015; The 1973), *Sr22a*/*Sr22b* (Gerechter-Amitai et al. 1971; Luo et al. 2022), *Sr35* (Saintenac et al. 2013), *Sr60* (Chen et al. 2018a), and *SrTm4* (Briggs et al. 2015). Except for the recessive gene *SrTm4*, all previously mapped *Sr* genes in *T. monococcum* have been cloned (Chen et al. 2018b, 2020; Luo et al. 2022; Saintenac et al. 2013; Steuernagel et al. 2016).

*SrTm4*, discovered in cultivated *T. monococcum* accession PI 306540, is a recessive resistance gene effective against all *Pgt* races tested, including race TTKSK (Briggs et al. 2015). Using two mapping populations, this gene was previously mapped within a 2.1 cM interval on the distal region of chromosome arm 2A^m^L (Briggs et al. 2015). The objectives of this study were to: (1) obtain an *SrTm4* monogenic line; (2) generate a precise map of *SrTm4*; and (3) identify candidate genes within the physical maps of the *SrTm4* region.

## Material and methods

### Plant materials and mapping populations

*T. monococcum* accession PI 306540 was identified as having a unique resistance response to multiple *Pgt* races (Rouse and Jin 2011a, b), which was subsequently associated with the presence of four *Sr* genes: *Sr21*, *Sr60*, *Sr22b*, and *SrTm4* (Briggs et al. 2015; Chen et al. 2018b, 2020; Luo et al. 2022). PI 306540 was crossed with both wild *T. monococcum* ssp. *aegilopoides* accession G3116 (Dubcovsky et al. 1996) and cultivated *T. monococcum* ssp. *monococcum* accession PI 272557 (Rouse and Jin 2011b) to generate two segregating populations. Wild accession G3116 was selected as a susceptible parent because it is highly polymorphic compared to cultivated PI 306540 (Chen et al. 2018a; Dubcovsky et al. 1996). The other susceptible parent PI 272557 does not possess any known *Sr* genes (Rouse and Jin 2011b).

A high-density genetic map of *SrTm4* was constructed using 9522 recombinant gametes from the cross of G3116 × PI 306540. From cross PI 272557 × PI 306540, we selected two F_3_ families homozygous for the presence (TmR4-260; lacking *Sr21*, *Sr60*, and *Sr22b*) or absence (TmS4-110; carrying no *Sr* gene) of *SrTm4* using molecular markers. Markers *BQ461276* and *DR732348* flanking the *SrTm4* resistance gene (Briggs et al. 2015) were used to confirm the presence of the PI 306540 segment in the selected family, whereas diagnostic markers from cloned genes *Sr21* (Chen et al. 2018b), *Sr60* (Chen et al. 2020), and *Sr22b* (Luo et al. 2022) were used to determine absence of the other *Sr* genes. Finally, we explored the distribution of a chromosomal inversion within the *SrTm4* candidate region in a collection of 79 wild and cultivated *T. monococcum* accessions.

### Stem rust assays

Infection types of PI 306540, PI 272557, and G3116 to *Pgt* races TTTTF (isolate 01MN84A-1-2), TTKSK (04KEN156/04), TRTTF (06YEM34-1), MCCFC (59KS19), TPMKC (74MN1409), RKQQC (99KS76A-1), RCRSC (77ND82A-1), QTHJC (75ND717C), QFCSC (06ND717C), and SCCSC (09ID73-2) were reported in previous studies (Briggs et al. 2015; Rouse and Jin 2011a). Race TTTTF is virulent to resistance genes *Sr21*, *Sr60*, and *Sr22b*, but avirulent to *SrTm4* (Briggs et al. 2015; Chen et al. 2018a). Moreover, *SrTm4* showed a mesothetic resistant infection type (intermediate reaction with both resistant and susceptible infection types present) (Rouse and Jin 2011a).

In the current study, the parental lines, selected families TmR4-260 and TmS4-110, and segregating populations were evaluated with race TTTTF at the United States Department of Agriculture, Agricultural Research Service (USDA-ARS) Cereal Disease Laboratory. For plants carrying recombination events in the candidate gene region, we carried out progeny tests using at least 25 individuals from each F_2:3_ family with race TTTTF. We further confirmed the phenotypes of these critical lines in the next generation by challenging 25 F_3:4_ plants homozygous for the recombination with the same race.

We also evaluated the selected families TmR4-260 and TmS4-110 with the Chinese *Pgt* race 34C3RTGQM (isolate 20IAL32) at Peking University Institute of Advanced Agricultural Sciences. The virulence/avirulence formulae of the *Pgt* races are provided in Table S1. Plants were grown in growth chambers at 18 °C day/15 °C night with a 16 h light/8 h darkness photoperiod. Procedures for inoculation and scoring disease reactions were as described previously (Briggs et al. 2015; Stakman et al. 1962).

### RNA-seq and qRT-PCR analysis

From the population G3116 × PI 306540, we selected two F_4_ lines homozygous for the resistant *SrTm4* haplotype (R-F14 and R-K18) and two sister control lines (S-A13 and S-E14) carrying the susceptible haplotype. The selected lines and parental lines G3116 and PI 306540 (total replications = 3) were inoculated with *Pgt* race 34C3RTGQM and mock-inoculated with water in two independent chambers under the environmental conditions described above: 18 °C day/15 °C night and 16 h light/8 h darkness. Leaf samples from different plants were collected immediately before inoculation (0 h) and 3-, 6- and 14-days post inoculation (dpi).

Total RNAs were isolated using the Spectrum Plant Total RNA Kit (MilliporeSigma, MO, USA). We performed RNA-seq for *Pgt*-inoculated RNA samples of G3116 and PI 306540 and the selected F_4_ lines (R-F14, R-K18, S-A13 and S-E14) at 14 dpi (accession number PRJNA932462). RNA-seq library preparation and sequencing was carried out at Beijing Novogene Bioinformatics Technology Co., Ltd.. Sequencing data quality control, sequence alignment, and variant calling were performed as described previously (Jiang et al. 2023). Differentially expressed genes (DEGs) were identified using edgeR software (Robinson et al. 2010) with a false discovery rate (FDR) of 0.05. The significance of the differences in transcript levels between the two groups was estimated using Student’s *t*-tests. DEGs within the *SrTm4* candidate region of chromosome 2A were analyzed and a heatmap was created using the pheatmap package (Kolde and Kolde 2018). Principal component analysis (PCA) of RNA-seq data from the homozygous resistant lines (PI 306540, R-F14 and R-K18) and the susceptible lines (G3116, S-A13 and S-E14) was presented in supplemental Figure S1.qRT-PCR was carried out on an ABI QuantStudio 5 Real-Time PCR System (Applied Biosystems, CA, USA) using PowerUp SYBR Green Master Mix. Transcript levels were expressed as fold-*ACTIN* levels using the 2^−ΔCT^ method as described previously (Pearce et al. 2013).

### Development of molecular markers

Based on the RNA-seq data, single nucleotide polymorphisms (SNPs) between the two parental lines G3116 and PI 306540 spaced throughout the candidate gene region were selected to develop markers. The sequences flanking the target polymorphisms were obtained from the *T. aestivum* reference genome of ‘Chinese Spring’ (CS) RefSeq v2.1 (Zhu et al. 2021). Primers were designed using the software Primer3 web version 4.1.0 (https://primer3.ut.ee/) to amplify intronic regions carrying the target polymorphisms. PCR amplification products were sequenced using the Sanger method to confirm sequence polymorphisms between parents. The detected polymorphisms were used to develop Insertion-Deletion (InDel), or Cleaved Amplified Polymorphic Sequence (CAPS), or derived Cleaved Amplified Polymorphic Sequence (dCAPS) markers (Bhattramakki et al. 2002; Konieczny and Ausubel 1993; Neff et al. 1998).

### BAC library screening and sequencing

Bacterial artificial chromosome (BAC) libraries from *T. monococcum* accessions DV92 (Lijavetzky et al. 1999) and PI 306540 (Chen et al. 2020; Luo et al. 2022) were available at the Wheat Molecular Genetics Laboratory, University of California, Davis. High quality DNAs from the selected BAC clones were extracted using QIAGEN Large-Construct Kit (Qiagen, Hilden, Germany). BAC DNAs were fingerprinted using restriction enzyme *Hind*III. Selected BACs were sequenced using WideSeq at Purdue University (https://purdue.ilabsolutions.com/landing/808) or/and Illumina HiSeq 4000 platform at Beijing Novogene Bioinformatics Technology Co., Ltd.. We blocked the repetitive sequences in the *SrTm4* region using the *Triticeae* Repeat Sequence Database (http://wheat.pw.usda.gov/ITMI/Repeats/blastrepeats3.html), and searched the unblocked region using TBLASTX available at National Center for Biotechnology Information (NCBI, https://www.ncbi.nlm.nih.gov/).

### Statistical analyses

Genetic linkage maps were generated using MapChart 2.2 software (Voorrips 2002). The released reference genomes of diploid, tetraploid and hexaploid wheat varieties (Avni et al. 2017; Ling et al. 2018; Luo et al. 2017; Maccaferri et al. 2019; Walkowiak et al. 2020) were used in our analyses. The transcriptome databases of DV92 and G3116 (Fox et al. 2014) were also used to detect the expressions of candidate genes. The “Sorting Intolerant from Tolerant” (SIFT) algorithm was used to predict the effect of coding variants on protein function (Ng and Henikoff 2003). The assay for transposase-accessible chromatin (ATAC)-seq data from tetraploid wheat seedling roots was reported previously (Debernardi et al. 2022).

## Results

### Characterization of stem rust responses

**Fig. 1:**
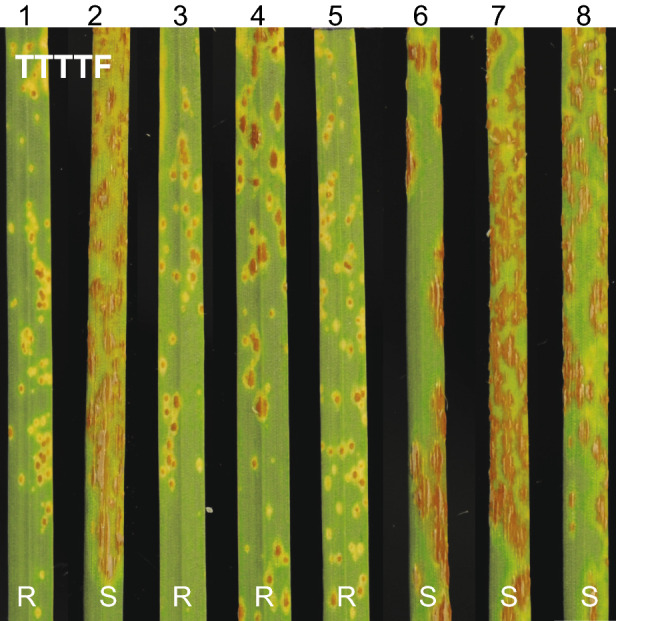
Stem rust reactions to *Pgt* race TTTTF (isolate 01MN84A-1-2) inoculated on leaves of segregating resistant and susceptible plants from cross G3116 × PI 306540. 1, PI 306540 (*SrTm4*); 2, G3116; 3–5, resistant F_3:4_ plants; 6–8, susceptible F_3:4_ plants. R, resistant; S, susceptible

A total of 388 F_2_ plants from cross PI 272557 × PI 306540 were used to separate *SrTm4* from the other three *Sr* genes (*Sr21*, *Sr60* and *Sr22b*) present in PI 306540. From this population, we selected F_3_ family TmR4-260 carrying only *SrTm4* and family TmS4-110 carrying no *Sr* gene. Seedlings from the TmR4-260 family exhibited mesothetic resistant infection types (ITs = ‘3’ to ‘31’) to *Pgt* races TTTTF and 34C3RTGQM, whereas seedlings from family TmS4-110 displayed susceptible infection types of ‘3+’ to ‘4’ (Fig. S2). *Pgt* race TTTTF, which is virulent on parental plants carrying resistance genes *Sr21*, *Sr60*, and *Sr22b*, but avirulent on plants carrying *SrTm4* (Briggs et al. 2015; Chen et al. 2018a), was used to determinate disease reactions in the G3116 × PI 306540 population. F_3:4_ seedlings homozygous for the presence of *SrTm4* showed ITs ranging from ‘;3’ to ‘31’ (similar to PI 306540), whereas plants homozygous for the absence of the gene displayed susceptible infection types (ITs = ‘3 + ’ to ‘4’, similar to G3116) (Fig. 1).

### High-resolution genetic map of *SrTm4*

The *SrTm4* locus was previously mapped within a 2.1 cM interval on chromosome arm 2A^m^L (Briggs et al. 2015). To accelerate the development of markers in the candidate region, we first performed an RNA-seq experiment to identify single nucleotide polymorphisms (SNPs) between parental lines G3116 and PI 306540. Approximately 40.9 million and 28.1 million PE150 reads were generated for PI 306540 and G3116, respectively. After removing low-quality reads and adaptors, ~ 94% of the reads were mapped to ‘Chinese Spring’ wheat reference genome RefSeq v2.1, and a total of 84,495 polymorphisms were identified.

**Fig. 2:**
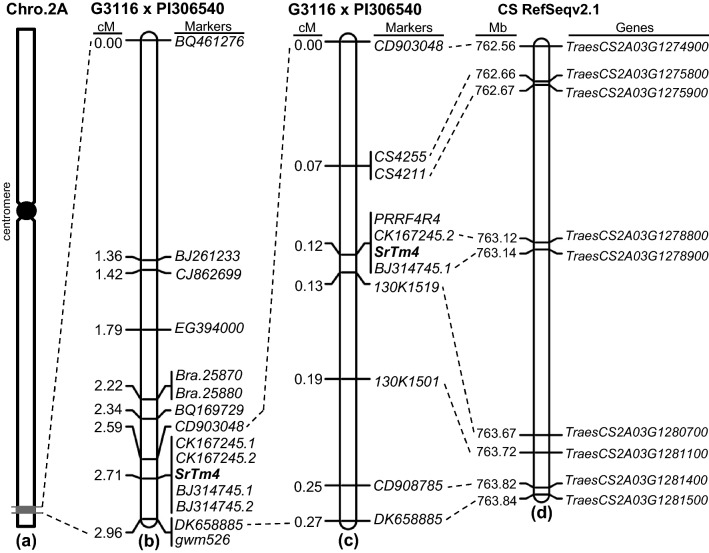
High-density genetic maps for stem rust resistance locus *SrTm4*. **a** Genomic region containing *SrTm4* (marked in gray) on the long arm of wheat chromosome 2A. **b** Genetic map based on 811 F_2_ plants and 14 molecular markers; **c** High-density genetic map based on 4761 F_2_ plants and 10 molecular markers; **d** Physical map of Chinese Spring. Coordinates are based on CS RefSeq v2.1

Based on these polymorphisms, we developed 12 new markers in the candidate region (Fig. 2b, Table S2). Screening of 811 F_2_ plants from the G3116 × PI 306540 cross yielded 48 plants with recombination events between *SrTm4*-flanking markers *BQ461276* (IWGSC RefSeq v2.1: 760,094,323 bp) and *gwm526* (763,867,267 bp), a genetic distance of 2.96 cM (Fig. 2b). The new markers were used to genotype the 48 lines with recombination events and to construct a genetic map of the *SrTm4* region (Fig. 2b). *SrTm4* was mapped within a 0.37 cM interval flanked by markers *CD903048* and *DK658885* and completely linked to markers *CK167245.1*, *CK167245.2*, *BJ314745.1* and *BJ314745.2* (Fig. 2b).

To define better the position of *SrTm4*, we screened another 3950 F_2_ plants with the new flanking markers *CD903048* and *DK658885*. The genetic distance between these two markers was estimated to be 0.27 cM based on 20 plants with informative recombination events identified in this screen plus another 6 plants found in the previous 811 plants. Using these informative recombination events and six new markers developed in this candidate region (Table S2), *SrTm4* was finally mapped within a 0.06 cM interval flanked by marker loci *CS4211* (IWGSC RefSeq v2.1: 762.67 Mb) and *130K1519* (IWGSC RefSeq v2.1: 763.67 Mb, Fig. 2c).

The candidate gene region partially overlapped with a 1.8 Mb chromosomal inversion between IWGSC RefSeq v2.1 (763.4 Mb to 765.2 Mb) and IWGSC RefSeq v1.1 (Fig. S3 and Table S3). Comparisons among published reference genomes of diploid, tetraploid and hexaploid wheat showed that this 1.8-Mb inversion is present only in IWGSC RefSeq v1.1 but absent in all other sequenced wheat varieties (Fig. S4), indicating that the IWGSC RefSeq v2.1 is the correct assembly for this region.

### Candidate genes for *SrTm4* within the colinear region of hexaploid wheat genome

The 0.06 cM candidate region between *SrTm4*-flanking markers *CS4211* and *130K1519* defines a 1.0-Mb region (762.67–763.67 Mb, Fig. 2d) in the reference genome of hexaploid wheat ‘Chinese Spring’ (IWGSC RefSeq v2.1) that contains 19 high-confidence annotated genes (*TraesCS2A03G1276000-TraesCS2A03G1280800*, Table S4).

Among these 19 candidate genes, four were differentially expressed (FDR < 0.05; *p*-value < 0.01; and |log2 foldchange|> 1) between the inoculated susceptible and resistant sister lines in the RNA-seq experiment (*TraesCS2A03G1276800*, *TraesCS2A03G1278700*, *TraesCS2A03G1280400*, and *TraesCS2A03G1280700*). Two of the differentially expressed genes (DEGs) showed that their transcripts were significantly higher in the plants carrying the susceptible *SrTm4* allele compared to the resistant allele, whereas the other two DEGs were downregulated in the same lines (Table S5).

Among DEGs identified in the RNAseq data (accession number PRJNA932462), 149 genes were significantly upregulated and 136 genes were significantly downregulated in the homozygous resistant lines (PI 306540, R-F14 and R-K18) relative to the susceptible lines (G3116, S-A13 and S-E14). Principal component analysis (PCA) of the RNAseq samples confirmed that the transcriptomes of the sister monogenic lines with and without *SrTm4* are very similar to each other and intermediate between the parental lines (Fig. S1). It also shows that the small number of DEGs detected between the sister lines are not sufficient to generate a clear difference between the two groups in the PCA.

### Physical maps of the *SrTm4* region

To determine whether additional genes were present in the candidate region in *T. monococcum*, we screened the BAC libraries of susceptible line DV92 and resistant parent PI 306540 using flanking markers *CS4211* and *130K1519* and three markers completely linked to *SrTm4* (*PRRF4R4*, *CK167245.2* and *BJ314745.1*, Fig. 2d). In addition, we developed new markers (Table S2) from genes located in the orthologous region in the ‘Chinese Spring’ reference genome to accelerate the screening process.

By chromosome walking, we identified 12 and 11 overlapping BACs from the BAC libraries of DV92 and PI 306540, respectively, covering the candidate gene region (Fig. S5 and S6). Based on sequencing and assembly of BAC sequences, we determined that the 0.06 cM candidate region defines a contiguous sequence of 754-kb region in *T. monococcum* accession PI 306540 (GenBank accession QQ503488). The 12 overlapping BACs of DV92 were sequenced at lower depth using the WideSeq approach, which yielded 30 non-overlapping contigs covering 652-kb excluding gaps.

### High confidence genes in the *SrTm4* candidate region

Our annotation of the 754-kb sequence showed no additional genes in the *SrTm4* candidate region in PI 306540 relative to Chinese Spring and DV92. *TraesCS2A03G1276100* is a pseudogene in both *T. monococcum* genotypes (DV92 and PI 306540), suggesting it is not a good candidate gene for *SrTm4*. For the other genes, we focused on those that were either differentially expressed or polymorphic in their coding regions between the resistant parent PI 306540 and the susceptible line DV92 (Table S5 and S6). We detected amino acid changes between PI 306540 and DV92 for nine expressed candidate genes (Table S6) and calculated SIFT scores to predict their effects on protein structure and function. Six genes had SIFT scores lower than 0.05, indicating high probabilities of deleterious effects (Table S6). Based on the functional annotation of these genes, we prioritized *TraesCS2A03G1276200*, *TraesCS2A03G1276800*, and *TraesCS2A03G1278900* given their known roles in plant defense against pathogens (Hopkins et al. 2008; Laluk et al. 2011; Qiu et al. 2021; Ruffel et al. 2002; Zhang et al. 2019a, 2021; Zhao et al. 2022).

The four DEGs identified in the candidate gene region are also potential candidates for *SrTm4* (Table S5). For each of these genes, we sequenced the promoter and open chromatin regions identified by ATAC-seq (Debernardi et al. 2022) (Fig. S7). We found multiple polymorphisms in these regions that may explain their differential expression.

Among the four DEGs, we eliminated *TraesCS2A03G1280400*, which is a short putative gene with a single predicted exon encoding a 120-amino acid peptide with no similarity to any known-function protein. Attempts to annotate the orthologous regions on chromosomes 2B and 2D revealed multiple frame-shift mutations, and no possible functional orthologs. Based on these results, we hypothesize that this is not a real gene, and we did not consider it further as a candidate for *SrTm4*.

**Fig. 3:**
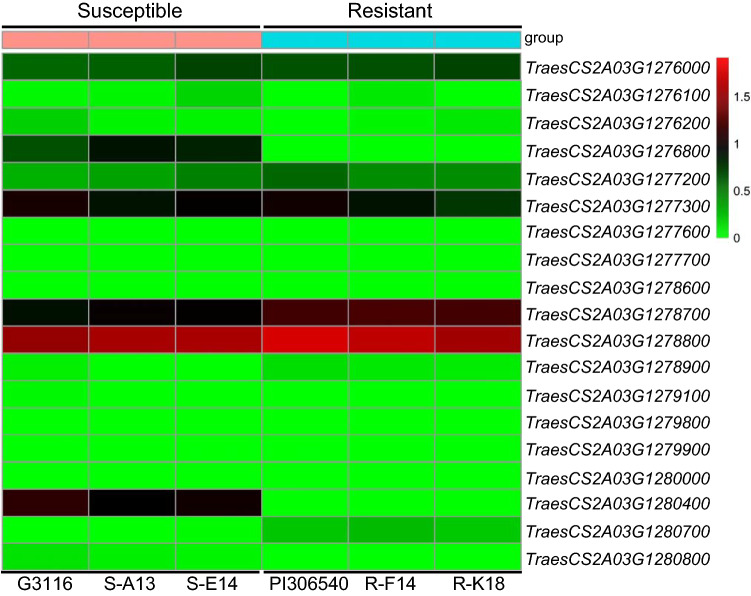
Transcript levels of high-confidence genes annotated in the candidate gene region. Differentially expressed genes (DEGs) between homozygous susceptible lines (G3116, S-A13 and S-E14) and homozygous resistant lines (PI 306540, R-F14 and R-K18) were identified using RNA-seq data. The heatmap was generated using the pheatmap package (Kolde and Kolde 2018)

The other three DEGs are annotated in the genome of Chinese Spring (RefSeq v2.1) as eukaryotic initiation factor 4A-III homolog B-like (*TraesCS2A03G1276800*), S-acyltransferase 11-like (*TraesCS2A03G1278700*), and acyl-activating enzyme 5 (*TraesCS2A03G1280700*). Their transcript levels were analyzed in *Pgt*-inoculated and mock-inoculated *T. monococcum* plants by qRT-PCR at 0 h, 3- and 6- days post inoculation (dpi). Since we were not able to detect transcripts of *TraesCS2A03G1276800* in PI 306540 and *TraesCS2A03G1280700* in G3116 based on RNAseq data (Table S5), their transcripts were evaluated only in PI 306540 or in G3116. We found that transcript levels of *TraesCS2A03G1276800* and *TraesCS2A03G1278700* in G3116 were significantly higher (*P* < 0.05) in *Pgt*-inoculated plants than in mock-inoculated controls only at 6 dpi (Fig. S8a, b). There was no significant difference in the transcript levels of *TraesCS2A03G1278700* and *TraesCS2A03G1280700* in PI 306540 between *Pgt*-inoculated and mock-inoculated plants (Fig. S8c, d). In addition, we also detected higher transcript levels of *TraesCS2A03G1278700* in PI 306540 than in G3116 (Fig. S8b, c), supporting the RNAseq data analysis (Table S5) (Fig. 3).

### Detection of a 593-kb chromosomal inversion in the candidate region that disrupts a potential candidate gene

**Fig. 4:**
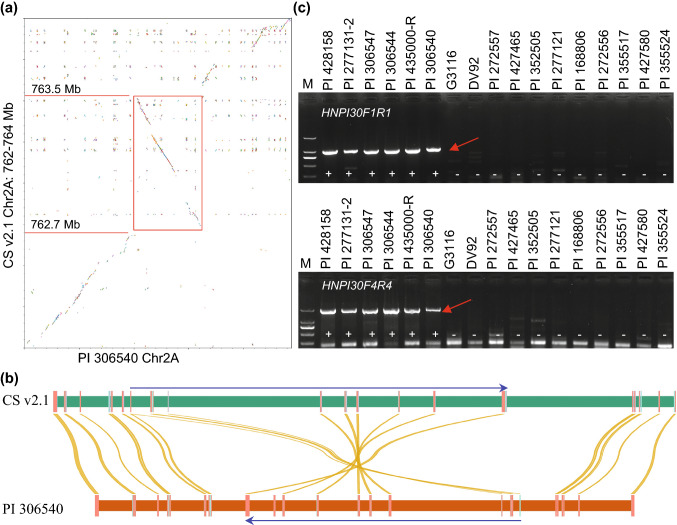
Chromosomal inversion in the candidate region. (**a**) Comparison of the *SrTm4* physical map in PI 306540 with the genomic sequence of Chinese Spring (RefSeqv2.1). The figure was generated using the Python drawing library matplotlib (Hunter 2007). The inverted region is highlighted by the red square. (**b**) Syntenic relationships between Chinese Spring and PI 306540. The figure was created using the NGenomeSyn program (https://github.com/hewm2008/NGenomeSyn). Blue arrows represent the inverted regions. (**c**) Dominant markers used to characterize the inversion breakpoints. Two dominant markers *HNPI30F1R1* and *HNPI30F4R4* (Table S2) were developed on the breakpoint junctions. These primers amplify PCR products of 1007-bp and 1859-bp when the 593-kb chromosomal inversion is present and no product when its absent. The amplification products are marked with a red arrow. + , PCR product present; -, no PCR product (colour figure online)

Based on chromosomal walking experiments, we observed that the order of the markers was reversed within a ~ 600-kb region between PI 306540 and DV92 (Fig. S5 and S6), indicating a potential chromosomal inversion in the candidate region. We then compared the two *SrTm4* physical maps with the Chinese Spring reference genome sequence (RefSeqv2.1) and confirmed the presence of an inverted segment in PI 306540 (Fig. 4a and b) relative to CS and DV92. Figure 4a shows that the PI 306540 inverted region was approximately 0.8 Mb, extending from 762.7 Mb to 763.5 Mb based on CS RefSeq v2.1 coordinates.

Further sequence analysis revealed the inversion breakpoints in PI 306540, as shown in Figure S9. On the proximal side, we delimited the inversion breakpoint to a 3.3-kb region in PI 306540. This 3.3-kb region includes the border of the inversion located at ~ 762.7 Mb (762,705,407–762,705,985 bp) and the inverted part located at ~ 763.5 Mb (763,515,576–763,513,146 bp) based on CS RefSeq v2.1 coordinates. The two parts of the sequences are located ~ 807 kb apart in the Chinese Spring reference genome but they are adjacent in the 3.3 kb region in PI 306540. On the distal side of the inversion, we also identified a 5.2-kb region in PI 306540 that contains two segments located far apart in Chinese Spring (762,705,509–762,704,291 bp and 763,515,638–763,518,339 bp, Fig. S9). Using the physical map of *SrTm4*, we were able to determine that the inverted region in PI 306540 was ~ 593 kb.

The proximal inversion breakpoint disrupted one gene, *TraesCS2A03G1276600LC.1*, which was annotated as encoding an L-type lectin-domain containing receptor kinase protein (designated here as *LLK1*). The inversion breakpoint is located in the coding region of this gene in PI 306540 and, therefore affects the protein structure and function. *TraesCS2A03G1276600LC.1* is a truncated gene in Chinese Spring since it carries premature stop codons, but its B- and D-genome homeologs encode complete proteins of 759 and 764 amino acids, respectively. Using the publicly available transcriptome databases of DV92 and G3116 (Fox et al. 2014) and other wheat genome sequences, we found that *LLK1* is expressed in susceptible *T. monococcum* DV92 and G3116, and encodes proteins containing ~ 763 amino acids in different wheat species (Fig. S10). Using qRT-PCR analysis, we found that the transcript levels of *LLK1* in G3116 were significantly higher in *Pgt*-inoculated than in mock-inoculated plants at both 3- and 6-dpi (*P* < 0.05; Fig. S11). *LLK1* was completely linked to *SrTm4* in the population of 4761 F_2_ plants, and is of particular interest because this type of protein was previously associated with disease resistance (Wang et al. 2015a, 2015b, 2018; Woo et al. 2016).

### Distribution of the chromosomal inversion

Based on BLASTN searches using the 3.3-kb and 5.2-kb segments carrying the inversion breakpoints in the published reference genomes, we determined that this chromosomal inversion was not present in sequenced accessions of *T. urartu* (G1812), *Aegilops tauschii* (AL8/78), *T. turgidum* subsp. *dicoccoides* (Zavitan), *T. turgidum* subsp. *durum* (Svevo), and *T. aestivum* (10 + wheat varieties in the Wheat Pan Genome project).

To characterize the distribution of the inversion in *T. monococcum*, we developed two dominant markers *HNPI30F1R1* and *HNPI30F4R4* (Table S2) on the breakpoint junctions. These two pairs of primers amplify PCR products of 1007-bp and 1859-bp fragments when the 593-kb chromosomal inversion is present and no product when it is absent (Fig. 4c). We also developed a dominant marker *DV92F1R1* (Table S2) for absence of the inversion. PCR amplification with primers *DV92F1R1* at an annealing temperature of 56 °C generates a 488-bp fragment when the inversion is absent and no amplification when it is present.

**Fig. 5:**
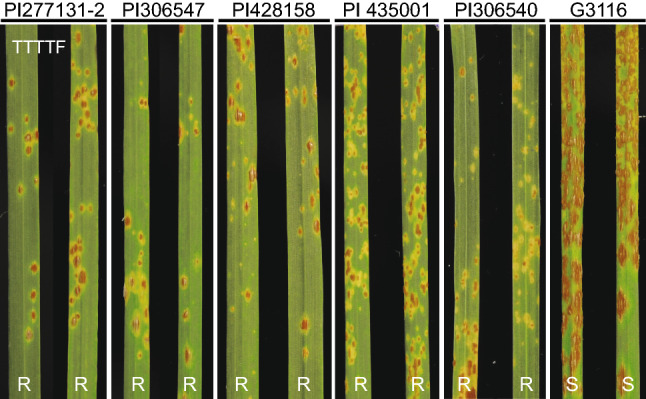
Infection types of *T. monococcum* accessions PI 277131-2, PI 306547, PI 428158, PI 435001, PI 306540, and G3116 in response to *Puccinia graminis* f. sp. *tritici* race TTTTF (isolate 01MN84A-1-2). Plants were grown in a growth chamber at 18 °C day/15 °C night with 16 h light/8 h darkness. R, resistant; S, susceptible

In a previous screen of 1,061 T*. monococcum* accessions using five selected *Pgt* races (Rouse and Jin 2011b), *SrTm4* was postulated to be present in five *T. monococcum* accessions in addition to PI 306540, including PI 352480, PI 306544, PI 355541, PI 435000-R, and PI 221414. We identified the same inversion breakpoints in these lines using the three dominant markers (Table S7). In addition, we evaluated a collection of 73 T*. monococcum* accessions and identified another four accessions (PI 277131-2, PI 306547, PI 428158, and PI 435001) where the same inversion was present based on the three dominant markers. The presence of the same inversion was confirmed by sequencing of the PCR products of *HNPI30F1R1* and *HNPI30F4R4*, which revealed identical sequences flanking the inversion as in PI 306540. Finally, we challenged these four lines with race TTTTF (isolate 01MN84A-1-2), and observed very similar responses to that conferred by PI 306540 (Fig. 5), although we cannot rule out the presence of additional or other *Sr* genes in these lines.

**Fig. 6:**
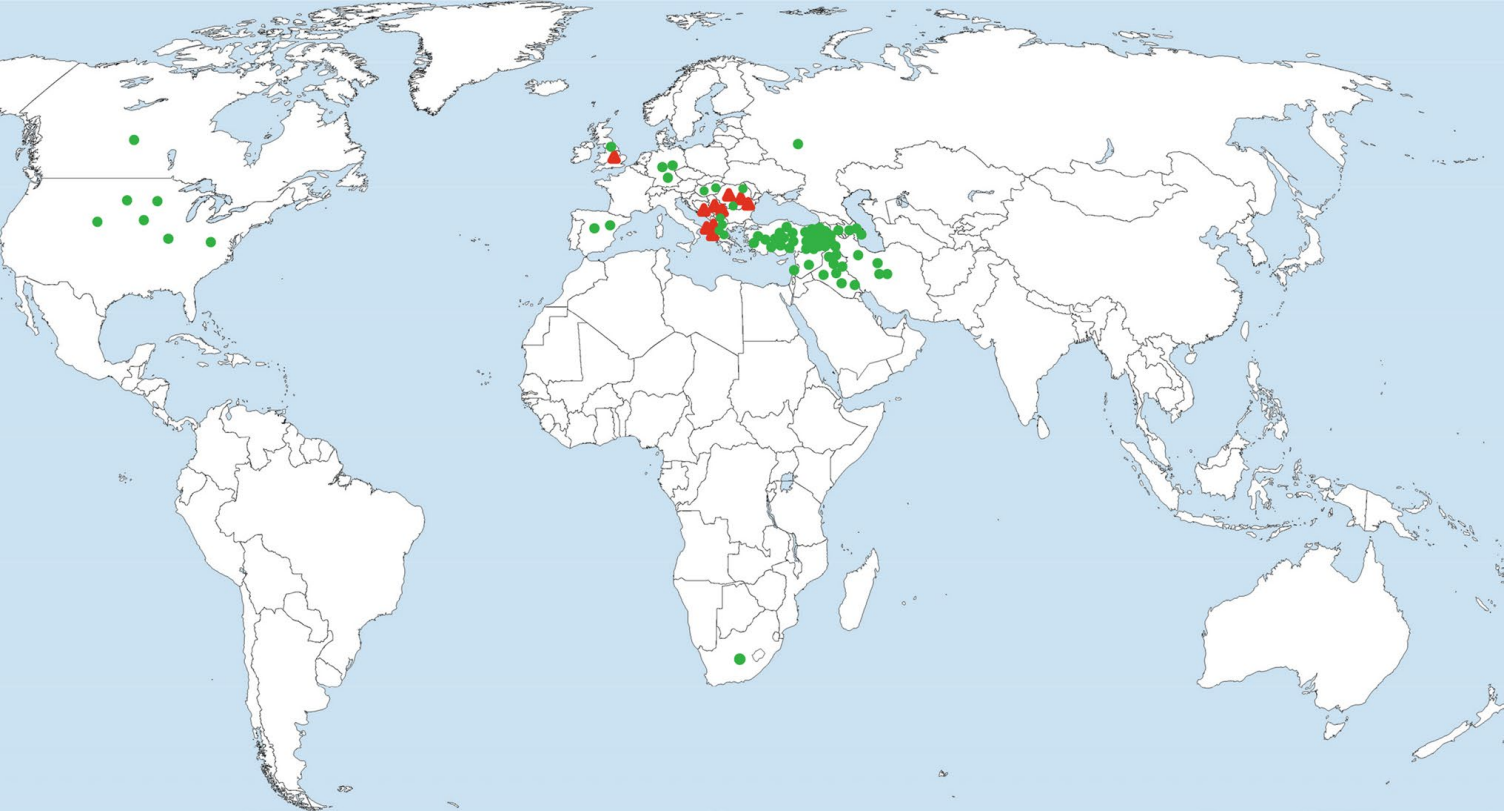
A collection of 79 *T. monococcum* accessions was used to test the presence/absence of the chromosomal inversion. Dominant markers *HNPI30F1R1*, *HNPI30F4R4*, and *DV92F1R1* (Table S2) were used to genotype these *T. monococcum* accessions. Green circles, accessions without the inversion; Red triangles, genotypes with the inversion (colour figure online)

In summary, the presence of the inversion seems to be linked to the *SrTm4* resistance allele. This inversion was found only in a few domesticated *T. monococcum* ssp. *monococcum* but was absent in all tested wild *T. monococcum* ssp. *aegilopoides* accessions (Table S7). Most of the accessions carrying the inversion were collected in the Balkans (Fig. 6), suggesting that the inversion event likely originated in this region.

## Discussion

### *SrTm4* shows broad-spectrum resistance to *Pgt* races

In this study, we generated *SrTm4* monogenic line TmR4-260 and sister susceptible line TmS4-110 lacking *SrTm4.* The monogenic line is useful to determine response profiles for *SrTm4* without confounding effects of other resistance genes*.* In a previous study, we showed that *SrTm4* conferred a low hypersensitive reaction to *Pgt* races TTKSK, TTTTF, TRTTF, QFCSC, MCCFC (Rouse and Jin 2011a), and mesothetic infection types for additional races TPMKC, RKQQC, RCRSC, and SCCSC that were virulent to *Sr21* (Briggs et al. 2015). Using the *SrTm4* monogenic line, we confirmed that *SrTm4* was effective against both North American and Chinese *Pgt* races (Fig. S2). The broad-spectrum resistance conferred by *SrTm4* makes it a potentially valuable genetic resource in breeding for resistance, especially to race Ug99 (TTKSK) and other more recently identified, widely virulent races.

### High-resolution mapping of *SrTm4* reveals an inversion linked to *SrTm4* resistance

Most plant disease resistance genes are dominant or partially dominant, but recessive *R* genes are also well documented. However, the molecular bases of recessive wheat stem rust recessive genes remain largely unknown, since all 18 *Sr* genes cloned so far in wheat and its relatives are dominant or partially dominant (Zhang et al. 2022). The recessive nature of *SrTm4* and the mesothetic resistant infection type provide increased incentive to clone this gene.

Using our high-resolution genetic map, the published reference genome of hexaploid wheat (The International Wheat Genome Sequencing Consortium 2018), and the available *T. monococcum* BAC libraries (Chen et al. 2020; Lijavetzky et al. 1999), we delimited the *SrTm4* candidate region to a 1.0-Mb region in common wheat Chinese Spring, a 652-kb discontinuous region in DV92, and a 754-kb continuous region in the resistant *T. monococcum* accession PI 306540.

A comparison of the 754-kb PI 306540 BAC sequence with the available genomic sequence of Chinese Spring and BAC sequence of DV92 revealed a 593-kb chromosomal inversion within the candidate region. Chromosomal inversions cause suppression of recombination, which likely explains the lack of recombination in the *SrTm4* candidate region. This inversion precluded a more detailed mapping of *SrTm4* in spite of the use of a very large mapping population (9522 gametes).

The geographic distribution of this inversion in the *T. monococcum* germplasm is limited to a few domesticated accessions that were collected mainly in the Balkans and all display similar mesothetic resistant responses to *Pgt* races (Fig. 5 and Table S7). Thus far, the presence of the inversion is completely linked to *SrTm4*.

Chromosomal inversions are important drivers of genome structure evolution in natural populations (Said et al. 2018). Inversions have the potential to disrupt genes at breakpoints, generate linkage blocks that cannot be broken by recombination, and cause positional effects on adjacent chromatin (Allshire et al. 1994; Spofford 1976). In this study, we observed significant gene expression differences in genes located close to the inversion breakpoint regions, such as *TraesCS2A03G1276800* and *TraesCS2A03G1280400* (Table S5), but we currently do not know if these differences are caused by position effects or disruption of the resistance gene.

### Candidate genes linked to *SrTm4*

We found no typical NLR gene within the candidate gene region. NLR genes are the most frequent gene class associated with pathogen resistance in wheat and other plant species (Li et al. 2021; Saintenac et al. 2013; Wang et al. 2022; Yang et al. 2022; Zhang et al. 2017, 2019b). We did not detect additional genes in the *SrTm4* candidate region in the susceptible *T. monococcum* line DV92 relative to the resistant parent PI 306540. However, we do not have a contiguous BAC sequence of DV92 and therefore cannot rule out the possibility that we missed the susceptibility gene located in the gap regions. However, this is unlikely because no additional gene(s) in the candidate region were found in Chinese Spring and other published wheat reference genomes.

Among the candidate genes, we identified six carrying predicted deleterious variants and four DEGs with polymorphisms in their regulatory regions (Table S5, S6), but we currently do not know if these changes affect their functions. Further functional characterization will be needed to demonstrate if one of these genes is *SrTm4*.

Except for the candidates described above, we also identified an L-type lectin-domain containing receptor kinase *LLK1*, that was completely linked to *SrTm4* and disrupted by an inversion in the resistant parent. Members of this gene family have been implicated in disease resistance in several plant species (Wang and Bouwmeester 2017; Wang et al. 2015b, 2018; Woo et al. 2016). Functional *LLK1* alleles are present in susceptible *T. monococcum* accessions but absent in all resistant *T. monococcum* accessions since the proximal inversion breakpoint disrupts its coding sequence. These results agree with the recessive nature of the resistance, and suggest that *LLK1* is a potential candidate for *SrTm4*. To determine if *LLK1* is the causal gene, we have initiated the development of loss-of-function mutations using both sequenced ethyl methane sulfonate (EMS)-mutagenized population of durum wheat Kronos (Krasileva et al. 2017) and CRISPR-Cas9 editing. If *LLK1* is demonstrated to be the causal gene for *SrTm4*, then the inversion itself will be the basis for the origin of *SrTm4*.

## Conclusions and practical implications

*SrTm4* is a broad-spectrum resistance gene and confers resistance to widely virulent *Pgt* races recently identified in the United States, Kenya, Yemen, and China (Table S1). Since *SrTm4* only confers intermediate levels of resistance when present alone, it would be necessary to combine it with other *Sr* genes to provide commercially useful levels of resistance. Pyramids of recessive and dominant resistance genes are expected to be an effective strategy for incorporating resistance (Pradhan et al. 2015). A combination of recessive and dominant *R* genes for resistance breeding has been reported in rice against bacterial blight pathogen (Li et al. 2001), in wild *Arachis* species against *Meloidogyne arenaria* (Church et al. 2005), in barley against leaf blotch (Garvin et al. 1997), and in pigeon pea against podfly and podborer (Verulkar et al. 1997).

Since the resistance genes *Sr21* (resistant haplotype R2 in PI 306540) and *SrTm4* are on the same chromosome arm located ~ 35 cM apart (Briggs et al. 2015; Chen et al. 2018b), it should be possible to introgress a *T. monococcum* chromosome segment carrying both genes into hexaploid wheat through the use of the *ph1b* mutation (Dubcovsky et al. 1995; Sears 1977). However, additional studies will be needed to test if the large introgressed *T. monococcum* segment with both *Sr* genes carries any undesirable genes. Even if *SrTm4* was successfully introgressed into hexaploid wheat, it may be required to also knock out the B- and D-genome homeologs to confer resistance given the recessive nature of *SrTm4*.

In summary, *SrTm4* recessive nature and its broad-spectrum resistance can make this gene a valuable component of gene pyramids combining different types of *Sr* genes. The high-density map of *SrTm4* and the tightly linked molecular markers identified in this study are useful tools to facilitate the cloning of this gene.

## Supplementary Information

Below is the link to the electronic supplementary material.

Supplementary file1 (PDF 1866 kb)

## Declarations

### Data availability statement

The BAC sequences and raw sequencing data reported in this study are available in the GenBank database as accession numbers QQ503488 and PRJNA932462. Data supporting the findings of this study are within the manuscript or the supplementary file.
