# Supplementary material for: High-resolution mapping of SrTm4, a recessive resistance gene to wheat stem rust
Source: Theor Appl Genet. 2023 Apr 27;136(5):120. doi: 10.1007/s00122-023-04369-z (PMC10140103; doi:10.1007/s00122-023-04369-z)
Supplement: Supplementary file 1 — Supplementary file1 (PDF 1866 kb) [file 122_2023_4369_MOESM1_ESM.pdf]

Supplementary Figures

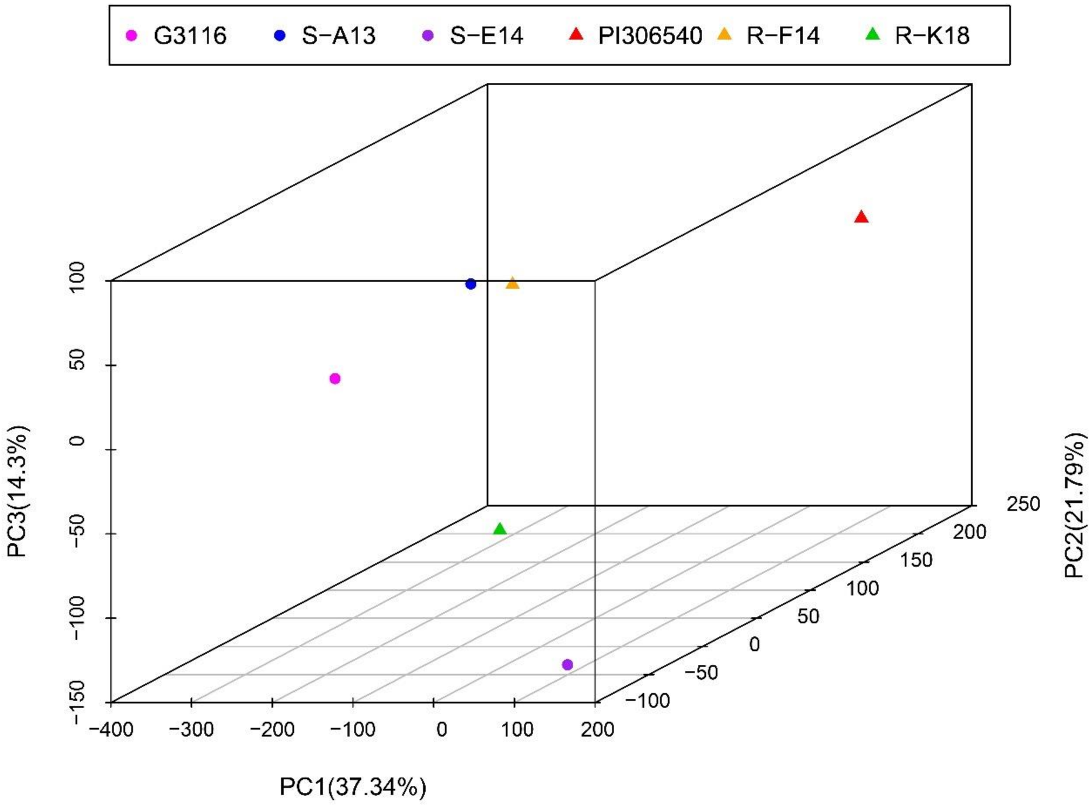

Supplementary Fig. S1 Principal component analysis (PCA) of RNA-seq data from homozygous resistant lines (PI 306540, R-F14 and R-K18) and susceptible lines (G3116, S-A13 and S-E14). Plants were inoculated with Chinese *Pgt* race 34C3RTGQM (isolate 20IAL32). Principal component analysis (PCA) was performed using the *prcomp* function from the R base package.

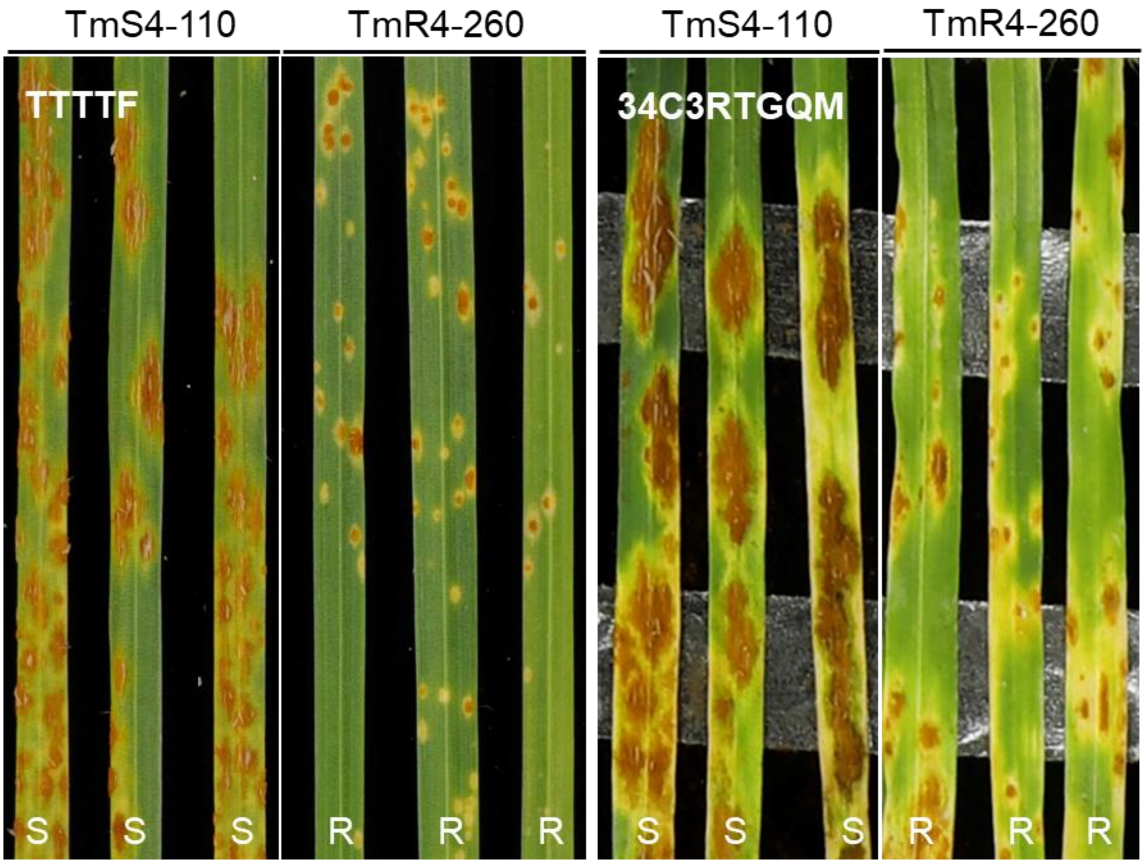

Supplementary Fig. S2 Reactions to *Pgt* races TTTTF (isolate 01MN84A-1-2) and 34C3RTGQM (20IAL32) in F_3 families TmS4-110 and TmR4-260. TmR4-260, selected F_3 family carrying only *SrTm4*; TmS4-110, F_3 family carrying no stem rust resistance gene. TmS4-110 and TmR4-260 were selected from cross PI 272557 × PI 306540. R, resistant; S, susceptible.

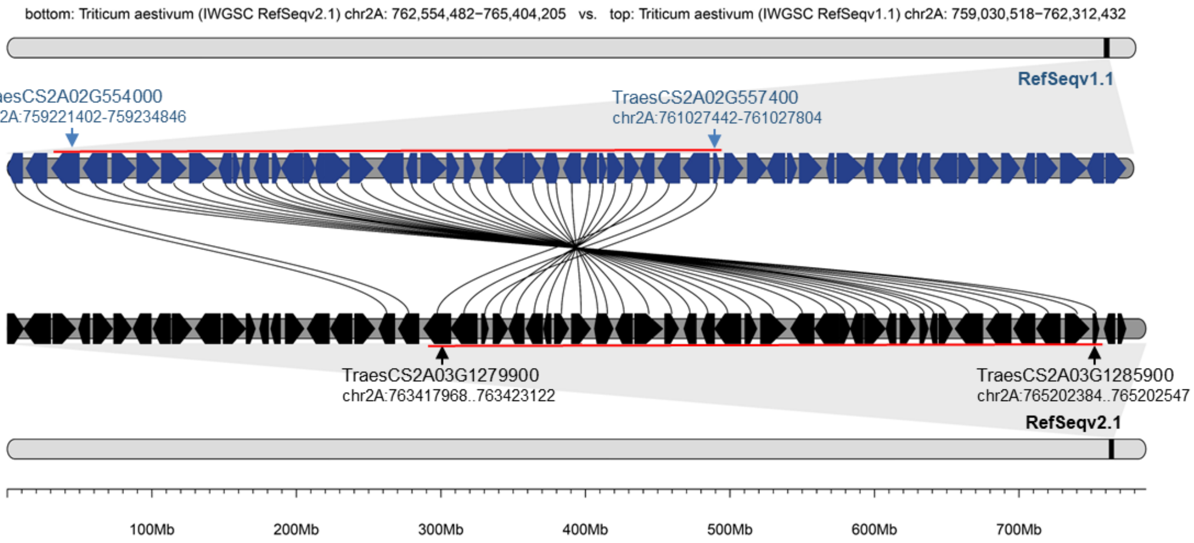

Supplementary Fig. S3 The comparison of IWGSC RefSeq v2.1 with IWGSC RefSeq v1.1 in the *SrTm4* candidate region. Red lines indicate the inverted regions between the two assembly versions. Genes are indicated by pentagons. Lines indicate similarity among genes. The Triticeae-GeneTribe database (http://wheat.cau.edu.cn/TGT/) (Chen et al. 2020) was used for gene pair analysis.

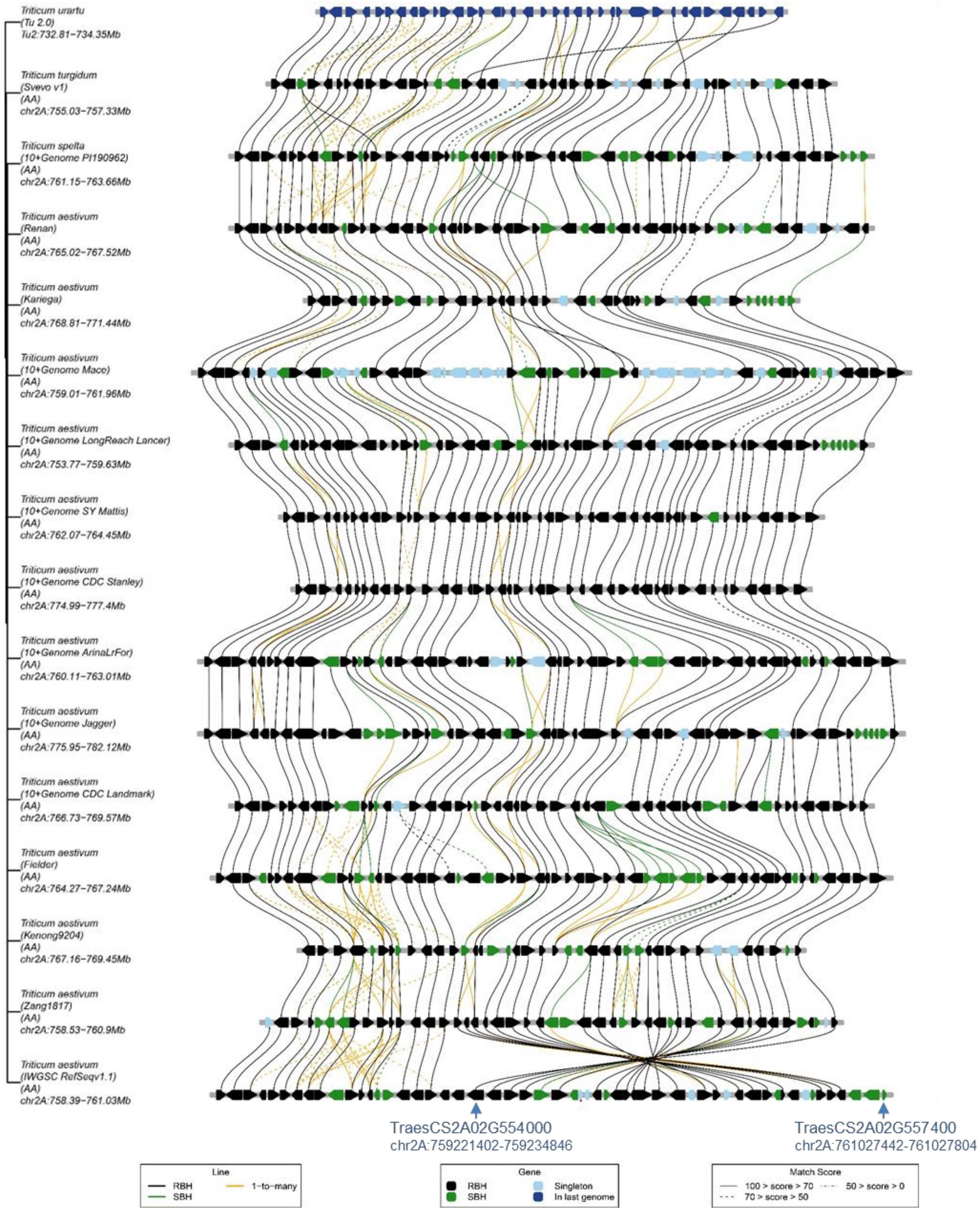

Supplementary Fig. S4 Micro-collinearity analysis of the *SrTm4* genetic region among different *Triticeae* genomes. Genes are indicated by pentagons. Lines indicate similarity among genes. This figure was produced using the Triticeae-GeneTribe database (http://wheat.cau.edu.cn/TGT/) (Chen et al. 2020).

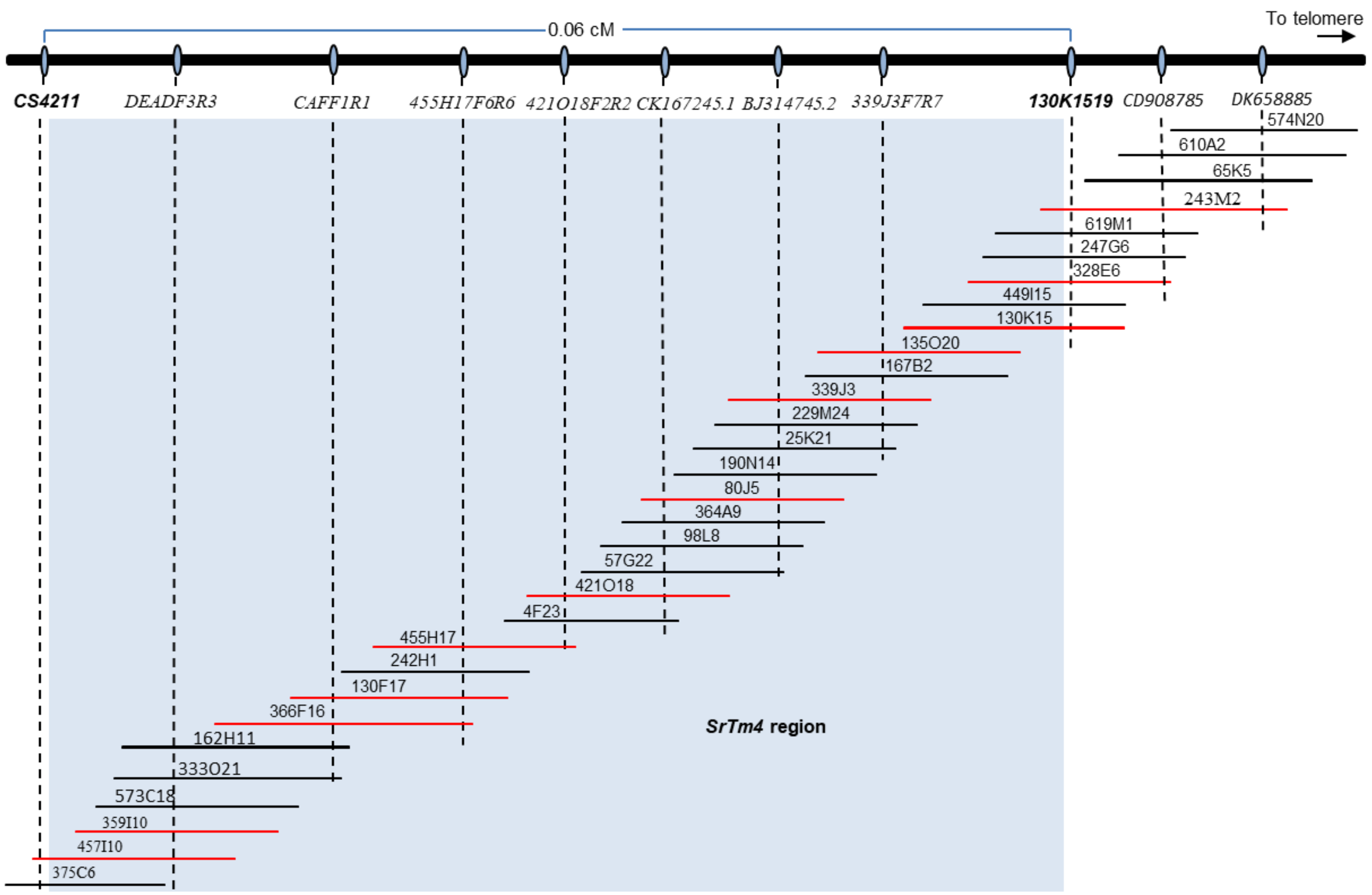

Supplementary Fig. S5 BACs from the *T. monococcum* accession DV92 in the *SrTm4* region. All BACs were screened from the DV92 BAC library and fingerprinted with restriction enzyme *Hind*III. Markers *CS4211* and *130K1519* delimit a candidate region for *SrTm4* (blue shaded square). The sequenced BACs are indicated in red lines.

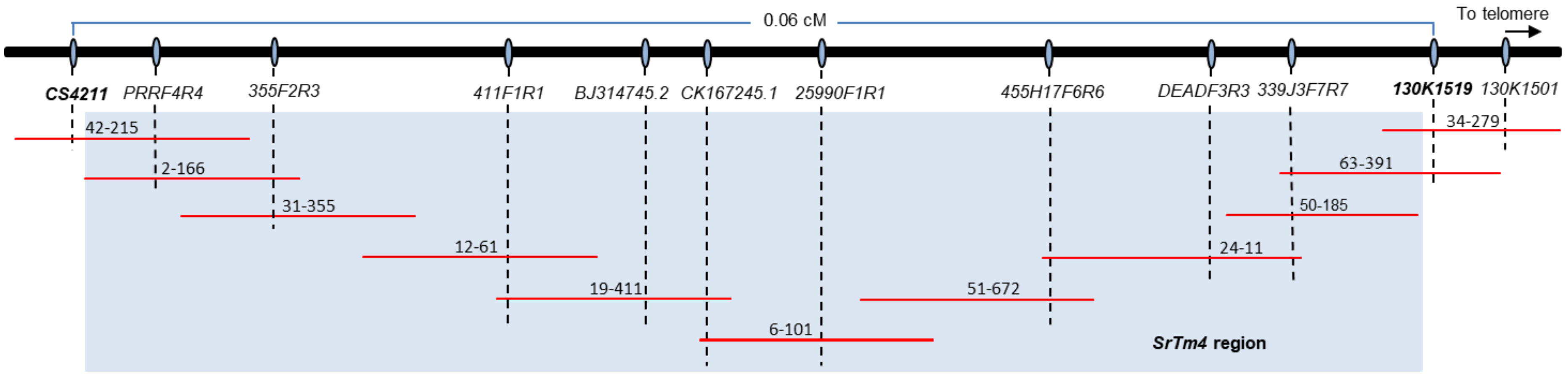

Supplementary Fig. S6 BACs from the *T. monococcum* accession PI 306540 in the *SrTm4* region. The non-gridded BAC library from PI 306540 was available at the Wheat Molecular Genetics Laboratory, University of California, Davis. Markers *CS4211* and *130K1519* delimit a candidate region for *SrTm4* (blue shaded square). The sequenced BACs are indicated by red lines.

TraesCS2A03G1276800:

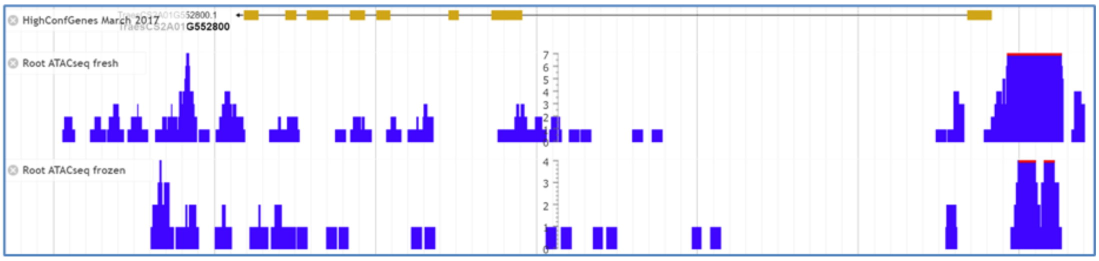

TraesCS2A03G1278700:

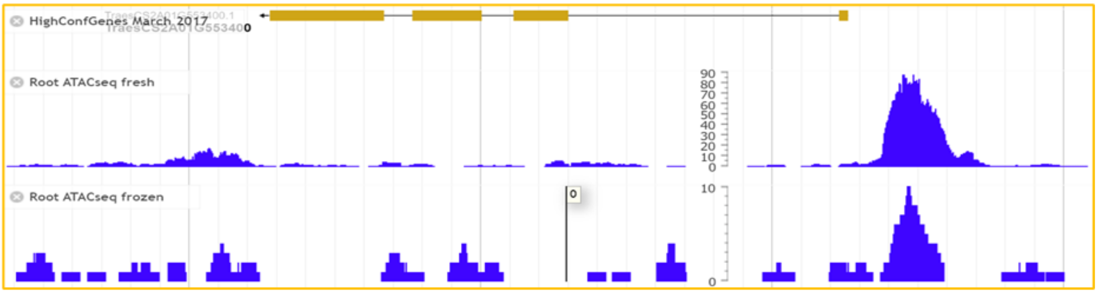

TraesCS2A03G1280400:

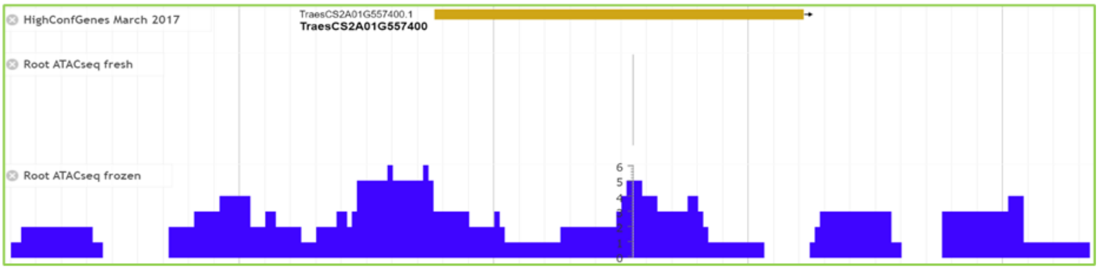

TraesCS2A03G1280700:

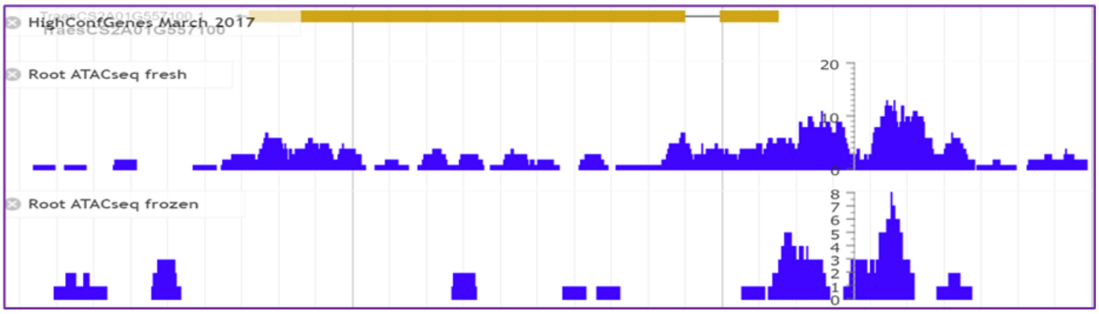

Supplementary Fig. S7 Promoter and other regulatory regions of the four differentially expressed genes identified by ATAC seq (Debernardi et al. 2022). The data were from tetraploid wheat seedling roots. We performed Sanger sequencing and found polymorphisms in the regulatory regions of all four genes.

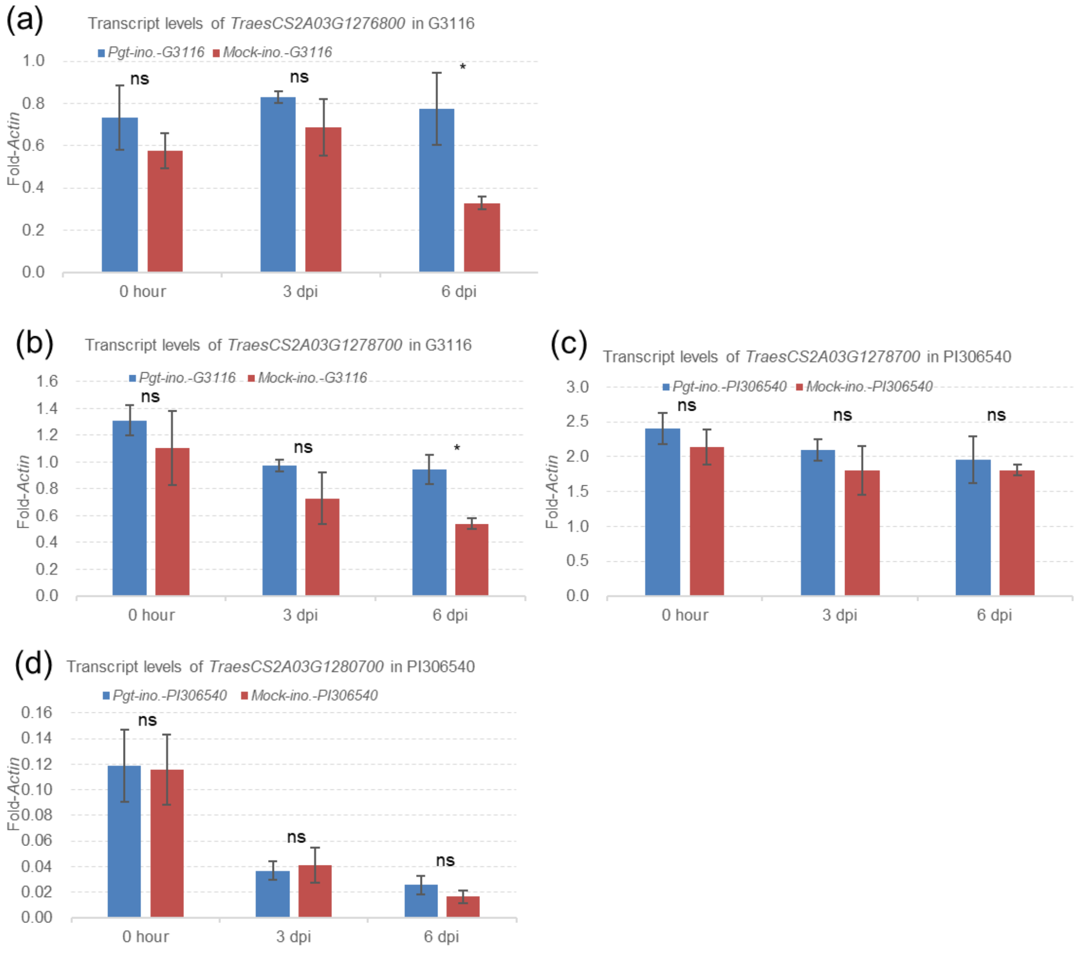

Supplementary Fig. S8 Transcript levels of *TraesCS2A03G1276800* (a), *TraesCS2A03G1278700* (b, c), and *TraesCS2A03G1280700* (c) in *Pgt*-inoculated and mock-inoculated *T. monococcum* plants. Leaves were collected from G3116 or PI 306540 at three time points: 0 hour, 3 dpi, and 6 dpi. Plants were grown in growth chambers at 18 °C day/15 °C night with 16 h light/8 h darkness and inoculated with race 34C3RTGQM. Transcript levels were expressed as fold-*Actin* (n = 3). Error bars are standard errors of the mean. ns, not significant; *, $P < 0.05$.

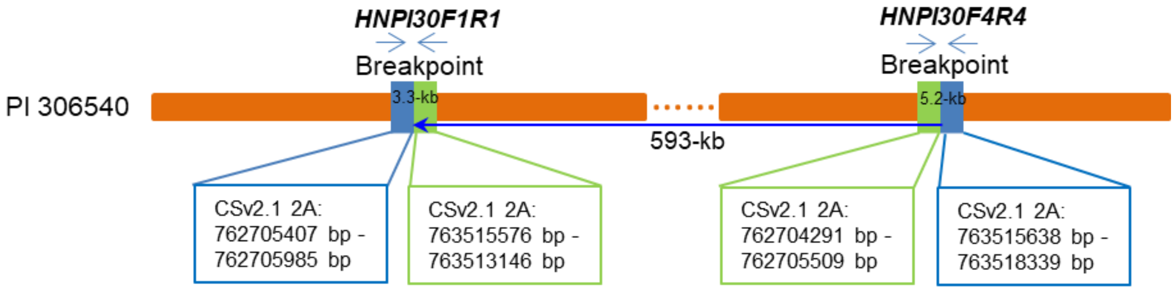

Supplementary Fig. S9 Inversion breakpoints in PI 306540. The physical positions of the 3.3-kb and 5.2-kb segments carrying the inversion breakpoints in the *T. aestivum* reference genome of Chinese Spring (RefSeq v2.1 coordinates). Two dominant markers *HNPI30F1R1* and *HNPI30F4R4* (Table S2) were developed on the breakpoint junctions.

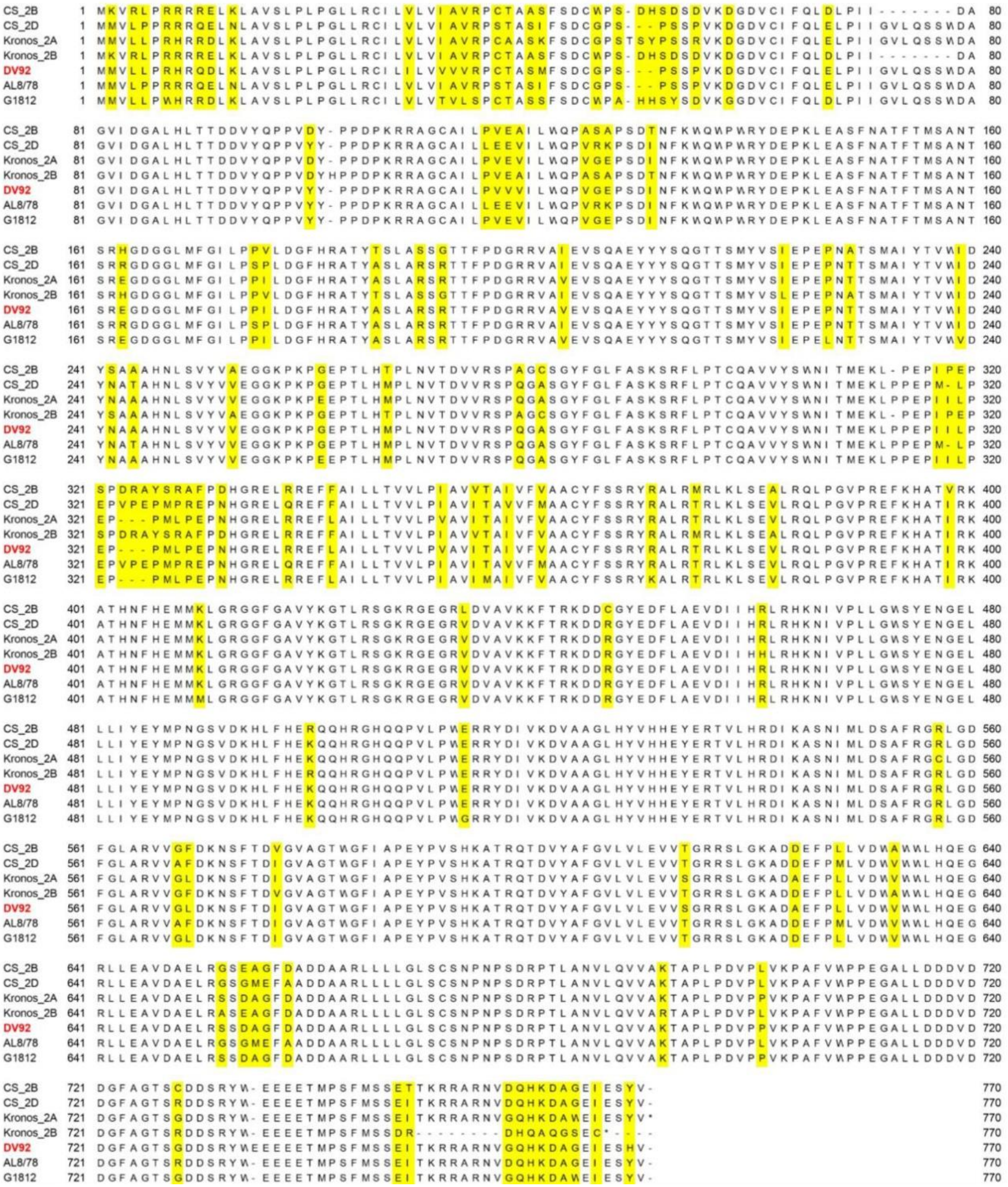

Supplementary Fig. S10 LLK1 protein sequences in accessions of different wheat species. The protein sequences are obtained from *T. monococcum* (DV92, A^mA^m), *T. urartu* (G1812, A^uA^u), *Aegilops tauschii* (AL8/78, DD), *T. turgidum* subsp. *durum* (Kronos, AABB) and *T. aestivum* (Chinse Spring, AABBDD). Protein polymorphisms among different haplotypes are highlighted in yellow.

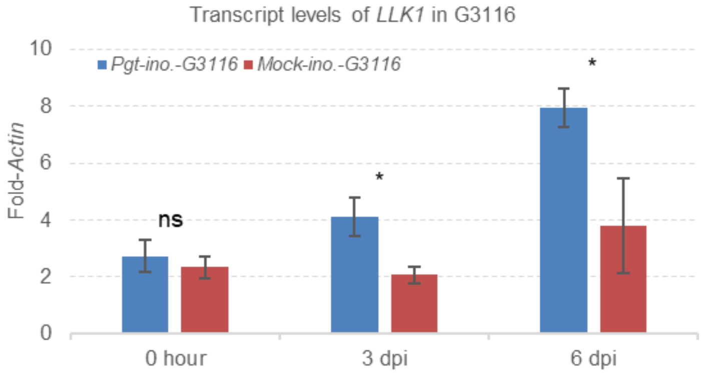

Supplementary Fig. S11 Transcript levels of *LLK1* in *Pgt*-inoculated and mock-inoculated *T. monococcum* plants. Leaves were collected from G3116 at three time points: 0 hour, 3 dpi, and 6 dpi. Plants were grown in growth chambers at 18 °C day/15 °C night with 16 h light/8 h darkness and inoculated with race 34C3RTGQM. Transcript levels were expressed as fold-*Actin* (n = 3). Error bars are standard errors of the mean. ns, not significant; *, $P < 0.05$.

Supplementary Tables

Table S1. Avirulence / virulence formulae of *SrTm4*-avirulent *Pgt* races used in the present study.

Race	Isolate	Origin	Avirulent to:	Virulent to:
TTTTF	01MN84A-1-2	USA	*Sr24 31*	*Sr5 6 7b 8a 9a 9b 9d 9e 9g 10 11 17 21 30 36 38 McN Tmp*
TTKSK	04KEN156/04	Kenya	*Sr21 24 36 Tmp*	*Sr5 6 7b 8a 9a 9b 9d 9e 9g 10 11 17 30 31 38 McN*
TRTTF	06YEM34-1	Yemen	*Sr8a 24 31*	*Sr5 6 7b 9a 9b 9d 9e 9g 10 11 17 21 30 36 38 McN Tmp*
MCCFC	59KS19	USA	*Sr6 8a 9a 9d 9e 9b 11 21 24 31 30 36 38*	*Sr5 7b 9g 10 17 McN Tmp*
TPMKC	74MN1409	USA	*Sr6 9a 9b 24 30 31 38*	*Sr5 7b 8a 9d 9e 9g 10 11 17 21 36 Tmp McN*
RKQQC	99KS76A-1	USA	*Sr9e 10 11 17 24 30 31 38 Tmp*	*Sr5 6 7b 8a 9a 9b 9d 9g 21 36 McN*
RCRSC	77ND82A-1	USA	*Sr6 8a 9e 11 24 30 31 38 Tmp*	*Sr5 7b 9a 9b 9d 9g 10 17 21 36 McN*
QTHJC	75ND717C	USA	*Sr7b 9a 9e 24 30 31 36 38 Tmp*	*Sr5 6 8a 9b 9d 9g 10 11 17 21 McN*
QFCSC	06ND717C	USA	*Sr6 7b 9b 9e 11 24 30 31 36 38 Tmp*	*Sr5 8a 9a 9d 9g 10 17 21 McN*
SCCSC	09ID73-2	USA	*Sr6 7b 8a 9b 11 24 30 31 36 38 Tmp*	*Sr5 9a 9d 9e 9g 10 17 21 McN*
34C3RTGQM	20IAL32	China	*Sr9e 10 12 13 15 17 19 20 23 27 30 31 33 35 36 37 38 Tmp*	*Sr5 6 7b 8a 9a 9b 9d 9g 11 14 18 21 24 25 28 29 32 34 McN*

Table S2. Primers used in the present study. CAPS, cleaved amplified polymorphic sequence; dCAPS, derived cleaved amplified polymorphic sequence; SSR, simple sequence repeat; InDel, insertion/deletion.

Marker	RefSeqv2.1 position (bp)*	Marker type	Forward primer (5' - 3')	Reverse primer (5' - 3')	Restriction enzyme	Annealing Tem. (℃)	Expected size (bp)#
BQ461276	760,094,323	InDel	GAGGAAACTTCAATGTGGC	CAGTAATGCTTATCGGGTAAC	–	50	1070
BJ261233	761,974,558	InDel	TCACAGGATCAATCATCAATACC	CTAGACCAGAAAAAAAGGGAAA	–	50	130
CJ862699	762,109,527	CAPS	GGCCGAACTTCGTTTGTTC	TTGCCTGCCTCGCTTTATA	*Dde*I	54	384
EG394000	762,267,160	InDel	GTGGATGGTAGAGGGGTTC	GTTGCGGATTGTCTGATAAG	–	52	840
Bra.25870	–	dCAPS	CGAATCGCATCCCAAACTCAACAG	ATCCCTTATACCCGTGGCTCATTT CACCA	*Bcc*I	53	178
Bra.25880	762,416,312	CAPS	CGTCAACCTCCTCCACATCA	TCCACGAGCCATTCCCTTC	*Hpy99*I	58	550
BQ169729	762,431,689	CAPS	AATGAGAGCAGGCAAAAGGGC	ACGACTGGGCGTCCAAGAAC	*SfaN*I	60	437
CD903048	762,561,106	CAPS	GTCCTCCCTTGTCCTGCTTC	GCTCCATTTGGCGATCTTTT	*Bcc*I	53	725
CS4255	762,663,267	CAPS	AGCCAGGACGGTCGCCACT	CCTTACGCCCATGTAATAGAGTC	*BsmA*I	60	543
CS4211	762,667,912	CAPS	GAATGCTAAACTCATACTCAA	CACTACAAATTATCCGAAAC	*Msl*I	52	872
PRRF4R4	762,688,156	CAPS	TGGAGGATGAGGGTGATGGTG	GCCGCTTTCTTCGGGTTCTT	*Bsr*I	57	729
CK167245.2	763,116,982	CAPS	TACTGCTTTCTTCAGGGGG	CGGTGGGTGCTGTTCTATC	*SfaN*I	52	448
BJ314745.1	763,140,786	CAPS	GACCAGCACGGAGGAAAAAT	GCAGAGGATGAGGAAGGCG	*BsmA*I	55	409
130K1519	763,669,364	InDel	CACGGGGTCACGCACCTCT	CACGGCACAACCACAACGAAT	–	60	1366
130K1501	763,718,794	CAPS	TCGGAGAGGGGAGGGCAGCA	CGACGAGCCAACCCAGGTGTCA	*Fok*I	62	601
CD908785	763,823,633	CAPS	TGGCACGTACTCAAACAAATCGC	CTCACCCCTGTCTCCCGCC	*Nae*I	60	453
DK658885	763,844,941	InDel	CGACAAGTCCCTCCAGTTCTTCG	TGTCACCGTCGCCCACCTT	–	61	506
gwm526	763,867,267	SSR	CAATAGTTCTGTGAGAGCTGCG	CCAACCCAAATACACATTCTCA	–	55	1064
DEADF3R3			TCTTCCCTCCATTCAAACCG	GCAAAATCACACATGCCACC	*NcoI-HF*	60	606
CAFF1R1			CGTTGGGATTGTGGAGAGAC	GATAAGGGCGACCTGGTTC	–	54	953
455H17F6R6			GTGGGGTCTCCTCTCCTTCT	TGCTTTTTCACCGTCTCGT	–	54	1155
421O18F2R2			GAGATTGTTTCTTGTCCCCT	ATAATGACCCAGCCCTTAG	–	52	972
CK167245.1			TCATTGCGTGAACGAGATA	ATACACTTGGGTTGTTGTGTG	–	51	595
BJ314745.2			AGGGAGAAAAGAGGCAAGA	CCGGTACGGAACCTATAAAG	*Apo*I	55	734
25990F1R1			GACGAGGATAGCATCAAGAAC	CGCCGAGAAGTCGAAGTAG	–	54	699
411F1R1			ACAGATGTCTAAAGACCACCG	TAAATTGTAAACTCAAGCGCC	–	55	1371
355F2R3			ATTAGTCTTGCGTCATCCAGC	AAGTTCTCCGACATCCTTTGG	–	55	1081
339J3F7R7			TGTACCTTATGTCAGTTTGCCCTT	TACCCCAGATTTGTGTCCCC	–	55	1380
HNPI30F1R1		dominant	CGTACCAAGAAGAGGGACAATG	GGTTGAGGAAGAAACGAAGACTG	–	57	1007
HNPI30F4R4		dominant	TCCACAGAGCCACCTACGCAT	GCCGACCCCACCGCTATTAC	–	57	1859
DV92F1R1		dominant	CTTCGGTTTATTCGCCTCAA	CGTAGCCACGGTCATCTTTC	–	56	488
DEADRF1R1		qRT-PCR	ATCCACGAGGTGCAAGCATT	ACTTTTGCCACCGACACAGG	–	60	126
LLKRTF1R1		qRT-PCR	GAGGTCGACATCATCCACCGG	TCGTAAGACCAACCAAGAAGAGGG	–	60	65
12787RF1R1		qRT-PCR	AGCAGTCACCCAAGGCAACC	GACCCCGCCACATATAACGA	–	60	133
12807RF1R1		qRT-PCR	GCTGCCCCTCCGTCGTCTA	CAGCACGGACACGACGTCG	–	60	125

*, Coordinates were based on CS RefSeq v2.1 (performed BLASTN searches using the forward primer sequences); #, expected size corresponding to the original size in PI 306540 without digestion.

Table S3. Comparison of IWGSC RefSeq v2.1 with IWGSC RefSeq v1.1 in the *SrTm4* candidate region. The 0.06 cM candidate region between *SrTm4*-flanking markers *CS4211* and *130K1519* are highlighted in bold. Inverted chromosomal regions between the two CS assembly versions are highlighted in yellow.

**Gene ID in CS RefSeqv2.1**	**Location (bp) in CS RefSeqv2.1**	**Gene ID in CS RefSeqv1.1**	**Location (bp) in CS RefSeqv1.1**
TraesCS2A03G1276000	**chr2A:762673482..762675402**	***TraesCS2A02G552500***	**chr2A:758514795-758517328(-)**
TraesCS2A03G1276100	**chr2A:762682913..762685170**	***TraesCS2A02G552600***	**chr2A:758524359-758526616(-)**
TraesCS2A03G1276200	**chr2A:762688659..762691833**	***TraesCS2A02G552700***	**chr2A:758530105-758533279(+)**
TraesCS2A03G1276800	**chr2A:762742964..762748775**	***TraesCS2A02G552800***	**chr2A:758553979-758559790(-)**
TraesCS2A03G1277200	**chr2A:762771517..762774846**	***TraesCS2A02G552900***	**chr2A:758583247-758587695(+)**
TraesCS2A03G1277300	**chr2A:762776599..762777469**	***TraesCS2A02G553000***	**chr2A:758588329-758589199(+)**
TraesCS2A03G1277600	**chr2A:762881352..762882176**	***TraesCS2A02G553100***	**chr2A:758692485-758693309(-)**
TraesCS2A03G1277700	**chr2A:762901014..762902246**	***TraesCS2A02G553200***	**chr2A:758712147-758713379(-)**
TraesCS2A03G1278600	**chr2A:763069201..763071878**	***TraesCS2A02G553300***	**chr2A:758880869-758883546(+)**
TraesCS2A03G1278700	**chr2A:763109468..763113895**	***TraesCS2A02G553400***	**chr2A:758921136-758925563(-)**
TraesCS2A03G1278800	**chr2A:763115994..763119996**	***TraesCS2A02G553500***	**chr2A:758927662-758931664(-)**
TraesCS2A03G1278900	**chr2A:763138869..763142537**	***TraesCS2A02G553600***	**chr2A:758950537-758954205(+)**
TraesCS2A03G1279100	**chr2A:763218851..763220925**	***TraesCS2A02G553700***	**chr2A:759030519-759032593(-)**
TraesCS2A03G1279800	**chr2A:763286359..763290090**	***TraesCS2A02G553900***	**chr2A:759098027-759101758(-)**
TraesCS2A03G1279900	**chr2A:763417968..763423122**	***TraesCS2A02G557200***	**chr2A:760854360-760858205(-)**
TraesCS2A03G1280000	**chr2A:763420540..763426932**	***TraesCS2A02G557300***	**chr2A:760917097-760923489(-)**
TraesCS2A03G1280400	**chr2A:763530885..763531247**	***TraesCS2A02G557400***	**chr2A:761027442-761027804(+)**
TraesCS2A03G1280700	**chr2A:763668421..763670413**	***TraesCS2A02G557100***	**chr2A:760774468-760776460(-)**
TraesCS2A03G1280800.1	**chr2A:763671999..763674048**	***TraesCS2A02G557000***	**chr2A:760770833-760772882(+)**
TraesCS2A03G1280900.1	chr2A:763681055..763683279	*TraesCS2A02G556900*	chr2A:760761602-760763826(+)
TraesCS2A03G1281000.1	chr2A:763685108..763685692	*TraesCS2A02G556800*	chr2A:760759189-760759773(+)
TraesCS2A03G1281100.1	chr2A:763718701..763720671	*TraesCS2A02G556700*	chr2A:760724210-760726180(-)
TraesCS2A03G1281200.1	chr2A:763748506..763751187	*TraesCS2A02G556600*	chr2A:760693694-760696375(-)
TraesCS2A03G1281300.1	chr2A:763821252..763821563	*TraesCS2A02G556500*	chr2A:760621850-760622161(+)
TraesCS2A03G1281400.1	chr2A:763822897..763824386	*TraesCS2A02G556400*	chr2A:760619027-760620516(-)
TraesCS2A03G1281500.1	chr2A:763842845..763845095	*TraesCS2A02G556300*	chr2A:760597948-760600198(+)
TraesCS2A03G1281600.1	chr2A:763858881..763878844	*TraesCS2A02G556200*	chr2A:760564199-760584162(-)
TraesCS2A03G1281700.1	chr2A:763891378..763893332	*TraesCS2A02G556100*	chr2A:760549882-760551836(-)
TraesCS2A03G1282000.1	chr2A:764131205..764132796	*TraesCS2A02G556000*	chr2A:760306892-760308483(+)
TraesCS2A03G1282200	chr2A:764316663..764318541	*TraesCS2A02G555900*	chr2A:760118983-760120874(+)
TraesCS2A03G1282300.1	chr2A:764318980..764325143	*TraesCS2A02G555800*	chr2A:760112396-760118557(+)
TraesCS2A03G1282500.1	chr2A:764410518..764412081	*TraesCS2A02G555700*	chr2A:760024043-760025606(-)
TraesCS2A03G1282700.1	chr2A:764472658..764478652	*TraesCS2A02G555600*	chr2A:759963887-759969879(-)
TraesCS2A03G1283100.1	chr2A:764605567..764610351	*TraesCS2A02G555500*	chr2A:759831892-759836676(+)
TraesCS2A03G1283200.1	chr2A:764611231..764616644	*TraesCS2A02G555400*	chr2A:759825599-759831012(+)
TraesCS2A03G1283300.1	chr2A:764618073..764619485	*TraesCS2A02G555300*	chr2A:759822758-759824170(-)
TraesCS2A03G1283400.1	chr2A:764620023..764621726	*TraesCS2A02G555200*	chr2A:759820517-759822220(+)
TraesCS2A03G1283600.1	chr2A:764624507..764628340	*TraesCS2A02G555100*	chr2A:759813903-759817736(-)

TraesCS2A03G1283700.1	chr2A:764629351..764630652	*TraesCS2A02G555000*	chr2A:759811591-759812892(+)
TraesCS2A03G1284000.1	chr2A:764708323..764710355	*TraesCS2A02G554900*	chr2A:759731756-759733788(-)
TraesCS2A03G1284600.1	chr2A:764848682..764849416	*TraesCS2A02G554800*	chr2A:759589091-759589825(-)
TraesCS2A03G1284700.1	chr2A:764856130..764856537	*TraesCS2A02G554700*	chr2A:759581970-759582377(+)
TraesCS2A03G1284800.1	chr2A:764861123..764863073	*TraesCS2A02G554600*	chr2A:759575434-759577384(-)
TraesCS2A03G1284900.1	chr2A:764885629..764895246	*TraesCS2A02G554500*	chr2A:759542841-759552458(+)
TraesCS2A03G1285300.1	chr2A:764947058..764952733	*TraesCS2A02G554400*	chr2A:759484917-759490592(+)
TraesCS2A03G1285400	chr2A:764972025..764976020	*TraesCS2A02G554300*	chr2A:759461630-759465625(+)
TraesCS2A03G1285500.1	chr2A:764980372..764985306	*TraesCS2A02G554200*	chr2A:759452125-759457059(+)
TraesCS2A03G1285800.1	chr2A:765128096..765133355	*TraesCS2A02G554100*	chr2A:759303759-759309018(-)
TraesCS2A03G1285900.1	chr2A:765202384..765202547	*TraesCS2A02G554000*	chr2A:759221402-759234846(-)

Table S4. Predicted genes within the *SrTm4* candidate region based on the genomic sequence of Chinese Spring (RefSeqv2.1). High-confidence genes are highlighted in bold.

Gene ID in CS RefSeqv2.1	Position (chr2A)		Gene annotation	Strand
	Start	End		
TraesCS2A03G1276000	**762.673482Mb**	**762.675882Mb**	**No conserved domain**	-
TraesCS2A03G1276100	**762.682913Mb**	**762.68517Mb**	**F-box-like**	-
TraesCS2A03G1276200	**762.688659Mb**	**762.691833Mb**	**Pentatricopeptide repeat-containing protein**	+
TraesCS2A03G1276300LC	762.700796Mb	762.70136Mb	No conserved domain	+
TraesCS2A03G1276400LC	762.70141Mb	762.701688Mb	Ribonuclease H protein	+
TraesCS2A03G1276500LC	762.70262Mb	762.703045Mb	No conserved domain	+
TraesCS2A03G1276600LC	762.704725Mb	762.705746Mb	Protein kinase domain	+
TraesCS2A03G1276700LC	762.740717Mb	762.741385Mb	No conserved domain	+
TraesCS2A03G1276800	**762.742964Mb**	**762.748775Mb**	**Eukaryotic initiation factor 4A-III homolog B-like**	-
TraesCS2A03G1276900LC	762.750543Mb	762.755854Mb	Unknown	+
TraesCS2A03G1277000LC	762.760005Mb	762.760442Mb	No conserved domain	-
TraesCS2A03G1277100LC	762.763709Mb	762.764146Mb	Unknown	+
TraesCS2A03G1277200	**762.771517Mb**	**762.775965Mb**	**Unknown**	+
TraesCS2A03G1277300	**762.776599Mb**	**762.777469Mb**	**Unknown**	+
TraesCS2A03G1277400LC	762.784592Mb	762.791248Mb	Unknown	+
TraesCS2A03G1277500LC	762.799791Mb	762.799997Mb	Plant transposon protein	+
TraesCS2A03G1277600	**762.881352Mb**	**762.882176Mb**	**Unknown**	-
TraesCS2A03G1277700	**762.901014Mb**	**762.902246Mb**	**Unknown**	-
TraesCS2A03G1277800LC	762.903768Mb	762.904142Mb	Unknown	+
TraesCS2A03G1277900LC	762.996725Mb	762.997501Mb	No conserved domain	-
TraesCS2A03G1278000LC	763.041484Mb	763.042335Mb	No conserved domain	-
TraesCS2A03G1278100LC	763.044713Mb	763.044835Mb	No conserved domain	+
TraesCS2A03G1278200LC	763.045202Mb	763.045477Mb	Ribonuclease H protein	+
TraesCS2A03G1278300LC	763.045484Mb	763.046353Mb	No conserved domain	+
TraesCS2A03G1278400LC	763.047889Mb	763.04811Mb	Unknown	-
TraesCS2A03G1278500LC	763.048459Mb	763.048779Mb	No conserved domain	-
TraesCS2A03G1278600	**763.069201Mb**	**763.071878Mb**	**OPT oligopeptide transporter protein**	+
TraesCS2A03G1278700	**763.109468Mb**	**763.113895Mb**	**Protein S-acyltransferase 11-like**	-
TraesCS2A03G1278800	**763.115994Mb**	**763.119996Mb**	**Cytosolic iron-sulfur (Fe-S) protein**	-
TraesCS2A03G1278900	**763.138869Mb**	**763.142537Mb**	**BTB/POZ domain-containing protein**	+
TraesCS2A03G1279000LC	763.217527Mb	763.21803Mb	No conserved domain	+
TraesCS2A03G1279100	**763.218851Mb**	**763.220925Mb**	**Unknown**	-
TraesCS2A03G1279200LC	763.221355Mb	763.232929Mb	Unknown	-
TraesCS2A03G1279300LC	763.222534Mb	763.223536Mb	Transposition	+
TraesCS2A03G1279400LC	763.22662Mb	763.226943Mb	Cysteine desulfurase 2	+
TraesCS2A03G1279500LC	763.257942Mb	763.258421Mb	Unknown	+
TraesCS2A03G1279600LC	763.276676Mb	763.27699Mb	Plant mobile domain	-
TraesCS2A03G1279700LC	763.281466Mb	763.281774Mb	Unknown	+

TraesCS2A03G1279800	**763.286359Mb**	**763.29009Mb**	**Unknown**	-
TraesCS2A03G1279900	**763.417968Mb**	**763.426766Mb**	**No conserved domain**	-
TraesCS2A03G1280000	**763.42054Mb**	**763.426932Mb**	**No conserved domain**	-
TraesCS2A03G1280100LC	763.42447Mb	763.424859Mb	No conserved domain	-
TraesCS2A03G1280200LC	763.512579Mb	763.513508Mb	No conserved domain	-
TraesCS2A03G1280300LC	763.527871Mb	763.528413Mb	No conserved domain	+
TraesCS2A03G1280400	**763.530885Mb**	**763.531247Mb**	**Unknown**	+
TraesCS2A03G1280500LC	763.608809Mb	763.609723Mb	No conserved domain	+
TraesCS2A03G1280600LC	763.666031Mb	763.666453Mb	No conserved domain	+
TraesCS2A03G1280700	**763.668421Mb**	**763.670413Mb**	**Probable acyl-activating enzyme 5**	+
TraesCS2A03G1280800	**763.671999Mb**	**763.674048Mb**	**Casparian strip membrane protein**	-

Table S5. Transcript levels of the high-confidence genes annotated in the candidate region. TPM (transcripts per million) is used to measure expression levels. Differentially expressed genes (FDR < 0.05; p-value < 0.01; and |log2 foldchange| > 1) are highlighted in bold.

Gene ID in CS RefSeqv2.1	Gene annotation	G3116	S-A13	S-E14	PI 306540	R-F14	R-K18	*t*-test
TraesCS2A03G1276000	No conserved domain	2.746	2.950	4.098	3.441	3.539	4.070	0.418
TraesCS2A03G1276100	F-box-like	0.060	0.090	0.432	0.000	0.197	0.000	0.399
TraesCS2A03G1276200	Pentatricopeptide repeat-containing protein	0.498	0.096	0.102	0.000	0.070	0.210	0.398
TraesCS2A03G1276800	**Eukaryotic initiation factor 4A-III homolog B-like**	**3.507**	**6.630**	**5.736**	**0.000**	**0.000**	**0.000**	**0.005**
TraesCS2A03G1277200	Unknown	0.960	1.239	1.926	2.681	1.662	1.641	0.239
TraesCS2A03G1277300	Unknown	10.094	6.710	8.434	9.435	6.744	4.602	0.433
TraesCS2A03G1277600	Unknown	0.000	0.000	0.000	0.000	0.000	0.000	-
TraesCS2A03G1277700	Unknown	0.000	0.000	0.000	0.000	0.000	0.000	-
TraesCS2A03G1278600	OPT oligopeptide transporter protein	0.000	0.000	0.000	0.000	0.000	0.000	-
TraesCS2A03G1278700	**Protein S-acyltransferase 11-like**	**6.969**	**8.728**	**8.314**	**14.698**	**15.502**	**15.189**	**0.000**
TraesCS2A03G1278800	Cytosolic iron-sulfur (Fe-S) protein	31.683	37.265	37.465	57.388	45.721	35.223	0.186
TraesCS2A03G1278900	BTB/POZ domain-containing protein	0.140	0.100	0.000	0.327	0.204	0.171	0.071
TraesCS2A03G1279100	Unknown	0.000	0.000	0.000	0.000	0.000	0.000	-
TraesCS2A03G1279800	Unknown	0.000	0.000	0.000	0.000	0.000	0.000	-
TraesCS2A03G1279900	No conserved domain	0.000	0.000	0.000	0.000	0.000	0.000	-
TraesCS2A03G1280000	No conserved domain	0.000	0.000	0.000	0.000	0.000	0.000	-
TraesCS2A03G1280400	**Unknown**	**12.237**	**8.169**	**9.334**	**0.000**	**0.000**	**0.000**	**0.001**
TraesCS2A03G1280700	**Probable acyl-activating enzyme 5**	**0.000**	**0.000**	**0.000**	**0.632**	**0.804**	**0.606**	**0.000**
TraesCS2A03G1280800	Casparian strip membrane protein	0.279	0.158	0.083	0.000	0.000	0.000	0.039

Table S6. Polymorphisms in candidate proteins between PI 306540 and DV92. Only the genes expressed in *Pgt*-infected leaves were analyzed and summarized. Sorting intolerant from tolerant (SIFT) scores lower than 0.05 indicate a high probability of deleterious effects (Ng and Henikoff 2003).

Gene ID in CS RefSeqv2.1	Identity	Polymorphism between PI306540 and DV92	SIFT score	Gene annotation
TraesCS2A03G1276000.1	287/288=99.7%	R250W	0.00	No conserved domain
TraesCS2A03G1276100	-	pseudogenes in DV92 and PI 306540	-	F-box-like
TraesCS2A03G1276200	540/550=98.2%	K56E	0.00	Pentatricopeptide repeat-containing protein
		E57K	0.00	
		S67P	0.33	
		E68D	0.63	
		G69A	0.38	
		A deletion in PI306540	-	
		Y188F	0.58	
		E273D	0.57	
		E344Q	0.16	
		F475L	1.00	
TraesCS2A03G1276800	402/405=99.3%	S141G	0.36	Eukaryotic initiation factor 4A-III homolog B-like
		T321M	0.04	
		P349L	0.00	
TraesCS2A03G1277200.2	415/494=84%	vary widely for the last 94 amino acids	-	Unknown
TraesCS2A03G1277300	123/126=97.6%	P16S	0.09	Unknown
		E27A	0.00	
		S100L	0.00	
TraesCS2A03G1278700	347/353=98.3%	M111I	0.80	Protein S-acyltransferase 11-like
		I142L	0.15	
		I183V	0.21	
		I206V	1.00	
		L228R	0.62	
		K316R	0.40	
TraesCS2A03G1278800	267/267=100%	100% identical	-	Cytosolic iron-sulfur (Fe-S) protein
TraesCS2A03G1278900	536/549=97.6%	A46V	0.26	BTB/POZ domain-containing protein
		E60D	0.44	
		T144A	0.41	
		T145A	0.52	
		H261R	0.88	

		D265E	1.00	
		A289T	0.54	
		Y414C	0.00	
		P490S	0.39	
		D 491 Deleted	0.00	
		L502M	0.15	
		L506R	0.37	
		A525V	0.08	
TraesCS2A03G1280400	120/120=100%	100% identical	-	Unknown
TraesCS2A03G1280700	556/557=99.8%	T93P	0.16	Probable acyl-activating enzyme 5
TraesCS2A03G1280800	200/201=99.5%	A72N	0.04	Casparian strip membrane protein

Table S7. Infection types of *T. monococcum* accessions used for testing the presence/absence of the chromosomal inversion. Rust reactions shown here were based on the previous study (Rouse and Jin 2011). PCR products obtained for the *T. monococcum* accessions were re-evaluated with race TTTTF (isolate 01MN84A-1-2). *T. m. m.*, *Triticum monococcum* subsp. *monococcum*; *T. m. a.*, *T. monococcum* subsp. *aegilopoides*.

Accession	With/without *SrTm4*	Species	Source	TRTTF	TTKSK	TTTTF	QFCSC	MCCFC	HNPI30 F1R1	HNPI30 F5R5	DV92F 1R1
PI 306540	(+)	*T. m. m*	Romania	;123Z	0;	;123Z	;1-	0	Yes	Yes	No
PI 306544	(+)	*T. m. m*	Romania	;123+Z	0;	3+/;123+Z	;1-	0;	Yes	Yes	No
PI 352480	(+)	*T. m. m*	Albania	;123+Z	0;	2+2++	;1-	0;	Yes	Yes	No
PI 355541	(+)	*T. m. m*	Albania	2++;	0;	2++	0;	0;	Yes	Yes	No
PI 435000-R	(+)	*T. m. m*	Yugoslavia	;123+Z	0;	123+Z	2	;	Yes	Yes	No
PI 221414	(+)	*T. m. m*	Yugoslavia	2+3;/;1/;123Z	0	;123Z	0;	0;	Yes	Yes	No
PI 277131-2	(+)	*T. m. m*	Albania	2-	;	13-	2	;1/;	Yes	Yes	No
PI 306547	(+)	*T. m. m*	Romania	2+3;/;123+Z	;	;13-	2-2	0;	Yes	Yes	No
PI 428158	(+)	*T. m. m*	United Kingdom	2+3;/;123+Z	;12-	;123Z	;1-	;1	Yes	Yes	No
PI 435001	(+)	*T. m. m*	Bosnia and Herzegovina	2+3;/;123+Z	;	123+Z	2	;	Yes	Yes	No
PI 418580	(-)	*T. m. a*	Azerbaijan	4	3+	4	;1LIF/2+3	4/;1	No	No	Yes
PI 487249	(-)	*T. m. a*	Syria	4	2+/3/12+Z	3+	4	;1	No	No	Yes
CItr 17671	(-)	*T. m. a*	Turkey	3	1/12Z	4	22+	;1	No	No	Yes
PI 427507	(-)	*T. m. a*	Turkey	3+	3	3	22+	1	No	No	Yes
PI 427580	(-)	*T. m. a*	Turkey	4	123Z	4	4	;1	No	No	Yes
CItr 17674	(-)	*T. m. a*	Iran	3+	2++	3+	22+	1	No	No	Yes
PI 560720	(-)	*T. m. m*	Turkey	3+	2-	4	3+	4	No	No	Yes
G3116	(-)	*T. m. a*	Lebanon	4	12+Z	3	4	-	No	No	Yes
PI 427464	(-)	*T. m. a*	Azerbaijan	4	2+3Z	3	22+	;1	No	No	Yes
PI 427444	(-)	*T. m. a*	Turkey	3+	22+Z	3+	22+	1	No	No	Yes
PI 352273	(-)	*T. m. a*	Asia Minor	4	12Z	4	4	1	No	No	Yes
PI 427465	(-)	*T. m. a*	Armenia	4	2+3Z	4	3+	2	No	No	Yes
CI 2433	(-)	*T. m. m*	Germany	3+	2+	3+	4	;1	No	No	Yes
PI 427498	(-)	*T. m. a*	Turkey	4	3	3	22+	22+	No	No	Yes
PI 277121	(-)	*T. m. a*	Germany	4	2+3Z	4	4	;1	No	No	Yes
CItr 13963	(-)	*T. m. m*	United States	3+	2+	4	4	;1	No	No	Yes
PI 427662	(-)	*T. m. a*	Iraq	4	3+	4	22+	33+	No	No	Yes
PI 427478	(-)	*T. m. a*	Turkey	3+	1/12Z	3	22+	;1	No	No	Yes
CItr 13964	(-)	*T. m. m*	United States	3+	3-	3+	4	;1	No	No	Yes
CItr 17655	(-)	*T. m. m*	United States	3+	3-	4	4	;1	No	No	Yes
CItr 17657	(-)	*T. m. m*	United States	3+	;	3+	4	0;	No	No	Yes
PI 168803	(-)	*T. m. m*	United States	3+	3-	4	4	;1	No	No	Yes
PI 168806	(-)	*T. m. m*	United States	3+	22+3Z	3+	4	;1	No	No	Yes
PI 190940	(-)	*T. m. m*	Spain	3+	3-	4	4	;1	No	No	Yes

PI 377668	(-)	*T. m. m*	Former Yugoslavia	4	;	3/4	4	0;	No	No	Yes
PI 503874	(-)	*T. m. m*	South Africa	3+	3+	3+	33+	;1	No	No	Yes
PI 272556	(-)	*T. m. a*	Hungary	3+	4	3	4	4	No	No	Yes
PI 272557	(-)	*T. m. m*	Hungary	3+	4	4	4	4	No	No	Yes
PI 190942	(-)	*T. m. m*	Spain	3+	1	3+	4	;1	No	No	Yes
PI 362610	(-)	*T. m. m*	Macedonia	3/4	;1-	3	3+	0;	No	No	Yes
CItr 14520	(-)	*T. m. m*	Canada	3+	2	3	3+	;1	No	No	Yes
PI 355517	(-)	*T. m. m*	Asia Minor	2	12-	2+	2-	;1	No	No	Yes
PI 355538	(-)	*T. m. m*	Balkans	3+	;1-	3	4	;1-	No	No	Yes
PI 427796	(-)	*T. m. a*	Iran	3+	1	4	33+	;1	No	No	Yes
PI 427808	(-)	*T. m. a*	Iran	4	1	4	4	;1	No	No	Yes
PI 427693	(-)	*T. m. a*	Iraq	4	12Z	4	4	;1	No	No	Yes
PI 427688	(-)	*T. m. a*	Iraq	4	2-	4	4	;1	No	No	Yes
PI 427603	(-)	*T. m. a*	Turkey	4	2+3Z	4	3+	;1	No	No	Yes
PI 427592	(-)	*T. m. a*	Turkey	4	12+Z	3	4	;1	No	No	Yes
PI 427555	(-)	*T. m. a*	Turkey	4	2	3	4	;1	No	No	Yes
PI 427545	(-)	*T. m. a*	Turkey	4	2/2+	4	4	;1	No	No	Yes
PI 427497	(-)	*T. m. a*	Turkey	4	22+Z	4	4	1	No	No	Yes
PI 427452	(-)	*T. m. a*	Turkey	4	12Z	3+	3	1	No	No	Yes
PI 355453	(-)	*T. m. a*	Asia Minor	4	2	3	4	1	No	No	Yes
PI 427484	(-)	*T. m. a*	Turkey	4	2/2+Z	4	3+	;1	No	No	Yes
PI 427527	(-)	*T. m. a*	Turkey	4	12+Z	3+	4	;1	No	No	Yes
PI 352270	(-)	*T. m. a*	Germany	4	2+3Z	4	4	1	No	No	Yes
PI 427451	(-)	*T. m. a*	Turkey	3+	3	3	2	1	No	No	Yes
PI 401412	(-)	*T. m. a*	Iran	3	3	3	22+	3+	No	No	Yes
PI 427488	(-)	*T. m. a*	Turkey	4	22+Z	3+	2	22+	No	No	Yes
PI 427510	(-)	*T. m. a*	Turkey	3+	3	3	22+	22+	No	No	Yes
PI 427476	(-)	*T. m. a*	Turkey	4	1	4	22+	;1	No	No	Yes
PI 277135	(-)	*T. m. m*	Albania	3+	;	3/3+	2	0;	No	No	Yes
PI 306527	(-)	*T. m. a*	Romania	4	3	3+	4	4	No	No	Yes
PI 306532	(-)	*T. m. a*	Romania	4	4	3+	4	4	No	No	Yes
PI 352274	(-)	*T. m. a*	Asia Minor	4	4	4	4	4	No	No	Yes
PI 427461	(-)	*T. m. a*	United Kingdom	4	4	4	4	4	No	No	Yes
PI 427835	(-)	*T. m. a*	Iraq	4	4	3	3+	3+	No	No	Yes
PI 427937	(-)	*T. m. a*	Iraq	4	3+	4	33+	4	No	No	Yes
PI 538540	(-)	*T. m. a*	Turkey	4	33+	4	4	4	No	No	Yes
PI 538540	(-)	*T. m. a*	Turkey	4	33+	4	4	4	No	No	Yes
PI 538546	(-)	*T. m. a*	Iraq	4	3+	4	4	3+	No	No	Yes
PI 538552	(-)	*T. m. a*	Iraq	4/3+	3+	4	3+	3+	No	No	Yes
PI 554519	(-)	*T. m. a*	Former Soviet Union	3+	3+	3+	4	4	No	No	Yes
PI 119422	(-)	*T. m. m*	Turkey	3	3+	3+	4	3+	No	No	Yes

PI 427450	(-)	*T. m. a*	Turkey	3+	3-	3+	22+	1+	No	No	Yes
PI 427661	(-)	*T. m. a*	Iraq	4	3	4	22+	33+	No	No	Yes
PI 427477	(-)	*T. m. a*	Turkey	4	12-Z	3+	22+	;1	No	No	Yes
PI 554480	(-)	*T. m. a*	Turkey	3+/3	3-	3	;1	;1+	No	No	Yes

‡X, mesothetic reaction, also described by '4,3,;' or ';1,2,3,'. LIF, low infection frequency with most leaves with infection type (IT) 0 with rare pustules.

References

Chen Y, Song W, Xie X, Wang Z, Guan P, Peng H, Jiao Y, Ni Z, Sun Q, Guo W (2020) A collinearity-incorporating homology inference strategy for connecting emerging assemblies in the Triticeae tribe as a pilot practice in the plant pangenomic era. Molecular Plant 13:1694-1708

Ng PC, Henikoff S (2003) SIFT: Predicting amino acid changes that affect protein function. Nucleic Acids Res 31:3812-3814

Rouse M, Jin Y (2011) Stem rust resistance in A-genome diploid relatives of wheat. Plant Dis 95:941-944
